# Macrophage Reprogramming with Anti‐miR223‐Loaded Artificial Protocells Enhances In Vivo Cancer Therapeutic Potential

**DOI:** 10.1002/advs.202202717

**Published:** 2022-10-31

**Authors:** Paco López‐Cuevas, Can Xu, Charlotte E. Severn, Tiah C. L. Oates, Stephen J. Cross, Ashley M. Toye, Stephen Mann, Paul Martin

**Affiliations:** ^1^ School of Biochemistry Biomedical Sciences Building University Walk University of Bristol Bristol BS8 1TD UK; ^2^ Centre for Protolife Research School of Chemistry University of Bristol Bristol BS8 1TS UK; ^3^ National Institute for Health Research Blood and Transplant Research Unit (NIHR BTRU) in Red Blood Cell Products University of Bristol Bristol BS34 7QH UK; ^4^ Wolfson Bioimaging Facility Biomedical Sciences Building University Walk University of Bristol Bristol BS8 1TD UK; ^5^ Max Planck Bristol Centre for Minimal Biology School of Chemistry University of Bristol Bristol BS8 1TS UK; ^6^ School of Materials Science and Engineering Shanghai Jiao Tong University Shanghai 200240 P. R. China

**Keywords:** cancer, inflammation, macrophages, microRNA‐223, neutrophils, protocells, zebrafish

## Abstract

Several immune cell‐expressed miRNAs (miRs) are associated with altered prognostic outcome in cancer patients, suggesting that they may be potential targets for development of cancer therapies. Here, translucent zebrafish (*Danio rerio*) is utilized to demonstrate that genetic knockout or knockdown of one such miR, microRNA‐223 (miR223), globally or specifically in leukocytes, does indeed lead to reduced cancer progression. As a first step toward potential translation to a clinical therapy, a novel strategy is described for reprogramming neutrophils and macrophages utilizing miniature artificial protocells (PCs) to deliver anti‐miRs against the anti‐inflammatory miR223. Using genetic and live imaging approaches, it is shown that phagocytic uptake of anti‐miR223‐loaded PCs by leukocytes in zebrafish (and by human macrophages in vitro) effectively prolongs their pro‐inflammatory state by blocking the suppression of pro‐inflammatory cytokines, which, in turn, drives altered immune cell‐cancer cell interactions and ultimately leads to a reduced cancer burden by driving reduced proliferation and increased cell death of tumor cells. This PC cargo delivery strategy for reprogramming leukocytes toward beneficial phenotypes has implications also for treating other systemic or local immune‐mediated pathologies.

## Graphical abstract

Leukocytes have remarkable cancer surveillance capacities and so reprogramming them away from cancer nurturing and toward cancer killing is a therapeutic aspiration. This study reports how intravenous (or direct) injection of miniature artificial protocells loaded with anti‐microRNA‐223 cargo can reprogram cancer‐associated macrophages in larval and adult zebrafish leading them to be more pro‐inflammatory and thus able to drive melanoma shrinkage.

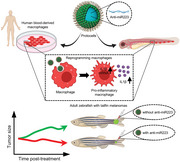

## 1 Introduction

Innate immune cells have a surveillance capacity which enables them to detect aberrant pre‐neoplastic cells arising at any tissue site in the body.^[^ ^1^, ^2^, ^3^, ^4^, ^5^, ^6^ ^]^ However, when innate immune cells encounter cancer cells they are often subverted by the cancer cells to nurture rather than destroy them.^[^ ^1^, ^6^, ^7^, ^8^ ^]^ Reprogramming inflammatory cells to clear early stage pre‐neoplastic clones or later tumor burden has been a cancer “immunotherapy” aspiration extending back to ancient bacteriotherapy anti‐cancer treatments. Indeed, serendipitous findings of Coley's in the early 1900s suggested that infection can enhance, or prime, the host immune response to better recognize and eradicate cancers.^[^ ^9^, ^10^ ^]^ In this regard, zebrafish larval studies have recently shown how exposure to infectious agents can activate the innate immune response to produce a reduction in cancer burden.^[^ ^11^ ^]^

Recent cancer immunotherapy breakthroughs in releasing adaptive immune cells from checkpoint inhibition have highlighted the therapeutic benefits of harnessing host immunity to eradicate cancers,^[^ ^12^ ^]^ and have led many to consider how innate immune cells might be reprogrammed to also disarm cancer cells. One group of potential targets for such reprogramming will include myeloid‐expressed miRs, since they clearly regulate leukocyte phenotype and behavior, and have been implicated in cancer progression. For example, several clinical studies have associated high levels of immune cell‐expressed microRNA‐223 (miR223) with poor prognostic outcome in a range of cancers, including both cutaneous and uveal melanoma, gastric carcinoma, and squamous cell carcinoma of head and neck.^[^ ^13^, ^14^, ^15^, ^16^, ^17^ ^]^ In addition, genetic knockout of miR223 in mouse and zebrafish reprograms leukocytes, increasing their pro‐inflammatory behavior particularly in a wound inflammatory setting.^[^ ^18^, ^19^ ^]^ Moreover, extensive sequence/evolutionary comparisons and expression studies indicate that miR223 is highly conserved between zebrafish and human, and thus, shares functions in both species,^[^ ^20^ ^]^ suggesting that zebrafish studies could potentially be directly translated into the clinic.

Here, we specifically target neutrophils and macrophages by virtue of their proclivity for phagocytosis and deliver reprogramming cargoes to them via intravenous (IV) or local injection of artificial cell‐like micro‐compartments (protocells [PCs]). We use non‐lipid proteinosome‐based PCs bounded by a cross‐linked protein–polymer semi‐permeable membrane which offer advantages over other potential cancer therapeutic delivery vectors in terms of stability and amenability for loading with high titers of guest cargoes.^[^ ^21^, ^22^, ^23^ ^]^ Cell‐sized therapeutic PCs, generated from erythrocyte membranes, have previously been injected intravascularly in rabbits, for systemic delivery of nitric oxide to blood vessels to induce vasodilation, and these PCs reach several major organs, including liver, spleen, and kidney, without any apparent tissue toxicity.^[^ ^24^ ^]^ However, PCs have not previously been tested for their in vivo capacity to deliver immunomodulatory cargoes to phagocytic cells. As a test cargo for reprogramming of innate immune cells, we chose to load PCs with single‐stranded 21/22‐nucleotide anti‐miR223 with the aim of inhibiting endogenous miR223 (miR223‐3p) activity. As anti‐miR223 freely diffuses through the proteinosome membrane (membrane cut‐off, ca. 65 kDa^[^ ^25^ ^]^), we pre‐loaded the proteinosomes with a positively charged polysaccharide, diethylaminoethyl‐dextran (DEAE‐dextran, 150 kDa) prior to addition of anti‐miR223 to the external solution. Subsequent diffusion of anti‐miR223 through the membrane gives rise to electrostatically mediated reversible complexation with the entrapped DEAE‐dextran such that high concentrations of the payload are captured and retained within the aqueous lumen of the PCs as a deliverable cargo.

In this study, we utilize the translucency and genetic tractability of zebrafish to first directly test whether global knockout of miR223 leads to reduced cancer progression. We show that genetic miR223 knockdown in either neutrophils or macrophages alone, or in both lineages together, results in suppression of cancer growth. We then investigate the feasibility of mimicking this anti‐cancer effect with transient, and thus potentially therapeutic, knockdown of miR223 by delivery of anti‐miR223 into leukocytes by phagocytosis of PC delivery vectors. We perform high resolution imaging of fluorescently tagged PCs loaded with, and without, anti‐miR223 cargoes as they circulate in vivo and are taken up by immune cells. We show that phagocytosis of the anti‐miR223‐containing PCs changes the cytokine expression and secretion profile and phenotypic state of immune cells as well as their behavior in the vicinity of cancer cells, in ways that far exceed injection of free anti‐miR223 alone. Overall, our results show the feasibility of using PCs to deliver cargoes for reprogramming innate immune cells in vivo and demonstrate how this approach can be used to drive immune cell suppression of cancer growth in zebrafish, as a first step toward developing novel innate immunotherapy anti‐cancer treatments.

## 2 Results

### 2.1 miR223KO Leads to Reduced Cancer Growth

**Figure 1:**
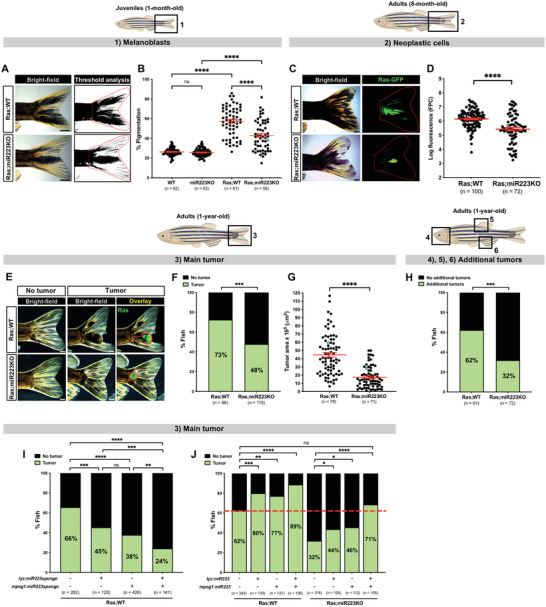
miR223 inhibition reduces cancer progression in zebrafish. A) Pigmentation of the tailfin quantified by threshold analysis of tail region (red dotted outline) in 1‐month‐old cancerous Ras;WT versus Ras;miR223KO juvenile fish. B) Dot plot showing percentage of pigmentation quantified from the regions imaged in (A) and (1). C) Ras‐GFP expression in the tail area (red dotted outline) of 8‐month‐old cancerous Ras;WT versus equivalent Ras;miR223KO adult fish. D) Dot plot showing levels of Ras‐GFP expression as quantified by fluorescent pixel count (FPC) from the regions imaged in (C) and (2). E) 1‐year‐old cancerous Ras;WT versus Ras;miR223KO adult fish bearing (or not) a tumor mass (red outline) on their tail. F) Bar chart showing percentage of cancerous fish with or without a tail tumor quantified from the regions imaged in (E) and (3). G) Dot plot showing tumor area quantified from the regions imaged in (E) and (3). H) Bar chart showing percentage of cancerous Ras;WT versus Ras;miR223KO adult fish bearing tailfin tumors, and any additional tumor (or not), quantified from the regions imaged in (4)–(6). See also Figure S1, Supporting Information. I) Bar chart showing percentage of 1‐year‐old cancerous adult fish with or without tumor, quantified from the regions imaged in (E) and (3), that are expressing miR223sponge in neutrophils (*lyz:miR223sponge*‐positive), macrophages (*mpeg1:miR223sponge*‐positive), or both lineages, in an otherwise Ras;WT background. See also Figure S2A–D, Supporting Information. J) Bar chart showing percentage of 1‐year‐old cancerous adult fish with or without tumor, quantified from the regions imaged in (E) and (3), that are overexpressing miR223 in neutrophils (*lyz:miR223*‐positive), macrophages (*mpeg1:miR223*‐positive), or both lineages, in Ras;WT or Ras;miR223KO backgrounds; red dashed line marks the percentage of control Ras;WT fish (*lyz:miR223*‐*mpeg1:miR223*‐double‐negative) with tumor. See also Figure S2E–H, Supporting Information. Accompanying schematics illustrate developmental stage (juvenile or adult) and imaged area (black outlined box) used for each experiment. Data are pooled from three independent experiments, and analyzed using one‐way ANOVA test with Bonferroni's multiple comparisons test (B), unpaired two‐sided Mann–Whitney test (D,G), or Fisher's exact test (F,H–J), ns *p* ≥ 0.05, *p < 0.05, **p < 0.01, ***p < 0.001, ****p < 0.0001. Graphs (B), (D), and (G) show mean ± SEM; each dot represents one fish and blue dots correspond to the representative images shown in the panels. *n* = number of fish. Scale bars = 1 mm.

miR223 is an anti‐inflammatory miR that is dysregulated in numerous inflammatory conditions and in several human cancers.^[^ ^13^, ^14^, ^15^, ^16^, ^17^, ^26^ ^]^ Pre‐clinical mouse models have shown that miR223 is highly expressed in myeloid cells during progression of breast cancer and melanoma metastasis.^[^ ^27^ ^]^ In the context of wound inflammation, deletion of this miR in mice leads to enhanced clearance of bacteria from infected wounds,^[^ ^18^ ^]^ and similarly miR223‐deficient zebrafish exhibit an augmented wound inflammatory response.^[^ ^19^ ^]^ In the current work, we crossed the previously established miR223 knockout (miR223KO) fish^[^ ^19^ ^]^ with a cancerous Tg(*kita:HRAS^G12V^‐GFP*) line (from here on referred to as Ras fish) that have a predisposition to develop melanoma, mainly in the adult tailfin, due to expression of a human mutant oncogene, HRAS^G12V^, fluorescently tagged with green fluorescent protein (GFP), in melanoblasts.^[^ ^1^, ^28^ ^]^ By comparison to cancerous wild‐type (WT) fish (Ras;WT), the cancerous miR223KO fish (Ras;miR223KO) exhibit reduced pigmentation of the tailfin at the juvenile stage (1‐month‐old) (**Figure** **1**A,B), suggesting that knockout of miR223 functions to inhibit the melanoblast proliferation, and pre‐cancer phenotype in juvenile fish. In 8‐month‐old adult fish, we observe reduced GFP fluorescence in the tailfins of Ras;miR223KO fish (Figure 1C,D), which serves as an indicator of numbers of Ras neoplastic cells. In 1‐year‐old Ras;miR223KO adult fish, we observe significantly fewer individuals with established tumors in their tailfins (Figure 1E,F) and tumor size is significantly smaller than in Ras;WT fish (Figure 1G), again indicating that miR223KO appears to repress cancer progression. Additionally, fewer Ras;miR223KO fish with tumors in their tailfins go on to develop “secondary” tumors at a distant location in the head, dorsal, or anal fins, where these tumors tend to predominate in this Ras melanoma line^[^ ^28^, ^29^ ^]^ (Figure 1H and Figure S1, Supporting Information), suggesting that tumors are less invasive in a miR223KO background.

Since miR223 is mainly expressed by innate immune cells (neutrophils and macrophages) and targets pro‐inflammatory mRNAs,^[^ ^18^, ^19^, ^26^, ^30^, ^31^ ^]^ we hypothesized that the “protective” phenotype observed in Ras;miR223KO fish might be mediated by innate immune cells. To test this, we developed two approaches. First, we inhibited miR223 by expressing competitive endogenous antisense RNA inhibitors (miR223 “sponges”) specifically in neutrophils of Ras;WT fish (Tg(*kita:HRAS^G12V^‐GFP;lyz:TagRFP‐miR223sponge*);WT) (Figure S2A,B, Supporting Information), or macrophages (Tg(*kita:HRAS^G12V^‐GFP;mpeg1:eGFP‐miR223sponge*);WT) (Figure S2C,D, Supporting Information), or in both of these lineages, and observed fewer 1‐year‐old fish with tailfin melanomas compared with control Ras;WT fish (Tg(*kita:HRAS^G12V^‐GFP;lyz:TagRFP;mpeg1:eGFP*);WT) without miR223 inhibition (Figure 1I). This was an additive effect such that knockdown of miR223 in both innate immune lineages resulted in the greatest reduction in tumor formation (Figure 1I). Second, we rescued miR223 levels in Ras;miR223KO fish, by expressing miR223 specifically in neutrophils (Tg(*kita:HRAS^G12V^‐GFP;lyz:miR223‐TagRFP*);miR223KO] (Figure S2E,F, Supporting Information), and/or macrophages (Tg(*kita:HRAS^G12V^‐GFP;mpeg1:miR223‐eGFP*);miR223KO) (Figure S2G,H, Supporting Information), and observed that miR223 overexpression in either neutrophils or macrophages of 1‐year‐old Ras;miR223KO fish leads to partial rescue of tumor incidence (Figure 1J). Only when miR223 was overexpressed in both innate immune lineages was the tumor incidence fully rescued, with a similar percentage of fish displaying tailfin melanomas to those of control Ras;WT fish (Tg(*kita:HRAS^G12V^‐GFP;lyz:TagRFP;mpeg1:eGFP*);WT) without miR223 overexpression (Figure 1J). When we overexpressed miR223 in neutrophils or macrophages or in both innate immune cell types, in Ras;WT fish, higher number of fish developed tumors by comparison to control Ras;WT fish (Figure 1J), further supporting the pro‐tumor role of miR223 in innate immune cells.

### 2.2 Injection of PCs Leads to Leukocyte‐Mediated Phagocytosis in Zebrafish Larvae

**Figure 2:**
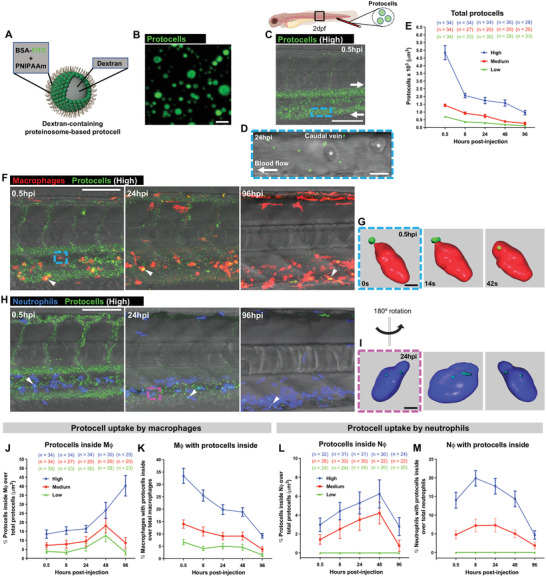
Systemically injected free‐circulating protocells are taken up by leukocytes. A) Schematic representation of dextran‐containing FITC‐labeled proteinosome‐based protocells. B) Single‐channel confocal image of FITC‐protocells. C) Multi‐channel confocal image of the flank of a 2 dpf casper larva after systemic injection of FITC‐protocells at 0.5 hpi; white arrows indicate the direction of the blood flow. D) High magnification view of (C) showing FITC‐protocells moving through the caudal vein at 24 hpi; red blood cells are also imaged (asterisks). See also Figure S3 and Movies S1 and S2, Supporting Information. E) Graph showing quantification of total protocells from the regions imaged in (C). F) Multi‐channel confocal images of the flank of Tg(*mpeg1:mCherry*) larvae after systemic injection of FITC‐protocells at 2 dpf and imaged at 0.5, 24, and 96 hpi, showing the distribution of protocells within macrophages (white arrowheads) and free within the vasculature. See also Figure S6 and Movies S7 and S8, Supporting Information. G) Imaris 3D reconstruction from confocal movie frames showing a macrophage engulfing and internalizing a free‐circulating FITC‐protocell within the caudal artery (blue dashed box in [F]). See also Movie S3, Supporting Information. H) Multi‐channel confocal images of the flank of Tg(*lyz:DsRed*) larvae after systemic injection of FITC‐protocells at 2 dpf and imaged at 0.5, 24, and 96 hpi, showing the distribution of protocells within neutrophils (white arrowheads) and free within the vasculature. I) Imaris 3D reconstruction from a confocal z‐stack image showing several FITC‐protocells internalized within a neutrophil at the CHT region (magenta dashed box in [H]). Rendered 3D image is rotated to demonstrate that protocells have been fully taken up by and reside within the neutrophil. See also Movie S4, Supporting Information. J–M) Graphs showing percentage of protocells within macrophages (J) or neutrophils (L), and percentage of macrophages (K) or neutrophils (M) containing protocell(s) quantified from the regions imaged in (F) and (H). See also Figures S4 and S5, and Movies S5 and S6, Supporting Information. “High”, “medium”, and “low” correspond to the different protocell titrations injected (1.25 × 10^7^, 5 × 10^6^, and 2.5 × 10^6^ protocells/µL, respectively). Accompanying schematics illustrate developmental stage (larva), type of injection (systemic), and imaged area (black outlined box) used for the experiments. Data are pooled from three independent experiments. Graphs show mean ± SEM, and each dot represents the mean of all fish analyzed. Mϕ = macrophages; *n* = number of fish; Nϕ = neutrophils. Scale bars = 2 µm (B), 100 µm (C,F,H), 10 µm (D), 5 µm (G,I).

Having demonstrated, using genetic approaches, how loss of miR223 in leukocytes can lead to a reduction of cancer progression in vivo, we next sought to develop a strategy for transient knockdown of miR223 in leukocytes. We planned to reprogram these cells using a potentially more therapeutically relevant viable system based on loading leukocytes with anti‐miR223 cargoes via PC delivery. We prepared dextran‐containing proteinosome‐based PCs by the spontaneous assembly and cross‐linking of bovine serum albumin‐NH_2_/poly(*N*‐isopropylacrylamide) (BSA‐NH_2_/PNIPAAm) nanoconjugates^[^ ^21^, ^22^, ^23^ ^]^ (**Figure** **2**A). PCs have been developed to have remote sensing,^[^ ^32^ ^]^ phagocytic and predatory behaviors,^[^ ^33^, ^34^ ^]^ chemical communication,^[^ ^25^, ^35^, ^36^ ^]^ and to host gene‐directed protein synthesis and enzyme catalytic reactions,^[^ ^37^ ^]^ among other features, in vitro, but have not been tested as potential delivery vectors of therapeutic agents to leukocytes in vivo.^[^ ^35^ ^]^ We reasoned that their optimal diameter for IV delivery of anti‐miR223 to immune cells of zebrafish larvae would be ≈2 µm to facilitate unhindered flow through the vasculature (since some vessel diameters are less than 5 µm^[^ ^38^ ^]^), and subsequent efficient phagocytosis by leukocytes. Thus, in our pilot studies, we systemically delivered fluorescently tagged 2 µm‐diameter dextran‐containing PCs, initially without anti‐miR223 cargo, by IV injection either via the Duct of Cuvier or the caudal vein of 2 days post‐fertilization (dpf) zebrafish larvae at increasing concentrations of proteinosomes suspended in media (Figure 2B–E). Injected PCs travel freely in a pulsatile fashion within the vasculature at a similar velocity to red blood cells (Figure 2C,D and Figure S3 and Movies S1 and S2, Supporting Information). By injecting PCs into larvae with fluorescently tagged monocytes/macrophages (Tg(*mpeg1:mCherry*)^[^ ^39^ ^]^), we investigated phagocytic uptake by macrophages (or monocytes while still circulating in vessels), and observe rapid, concentration‐dependent uptake of circulating PCs from minutes after injection and extending for a period of several days (Figure 2F,G,J,K). Peak uptake from an injected high concentration of 1.25 × 10^7^ PCs/µL, occurs by 0.5 h post injection (hpi), with more than 30% of macrophages having taken up PCs (Figure 2K). By 8 hpi, 15% of all PCs are within macrophages and less than 60% of PCs remain outside the macrophages by 96 hpi (Figure 2J). Real‐time phagocytic episodes were captured by video and typically took less than 30 s from macrophage‐PC contact to complete engulfment (Figure 2G and Movie S3, Supporting Information).

We undertook similar PC uptake studies in larvae with fluorescently tagged neutrophils (Tg(*lyz:DsRed*)^[^ ^40^ ^]^) after caudal vein injection (Figure 2H,I,L,M). Phagocytic uptake of the proteinosomes by neutrophils occurs but is less efficient than for macrophages (Figure 2H,I,L,M and Figure S4 and Movies S4–S6, Supporting Information), with 20% of the neutrophils containing PCs by 8 hpi at the highest injected concentration (Figure 2M). This is consistent with previous studies describing uptake of bacterial particles by macrophages versus neutrophils.^[^ ^41^ ^]^ Importantly, the numbers of macrophages and neutrophils remain identical to control media‐injected larvae after systemic injection of the anti‐miR223‐free PCs, indicating that the dextran‐containing proteinosomes alone do not trigger an inflammatory response or cell death in leukocytes (Figure S5, Supporting Information). We also observe limited uptake by endothelial cells, particularly in the larval caudal hematopoietic tissue (CHT) which may act as a “trap” for PCs, but in free‐flowing vessels we see almost no uptake of PCs (Figure S6 and Movies S7 and S8, Supporting Information).

**Figure 3:**
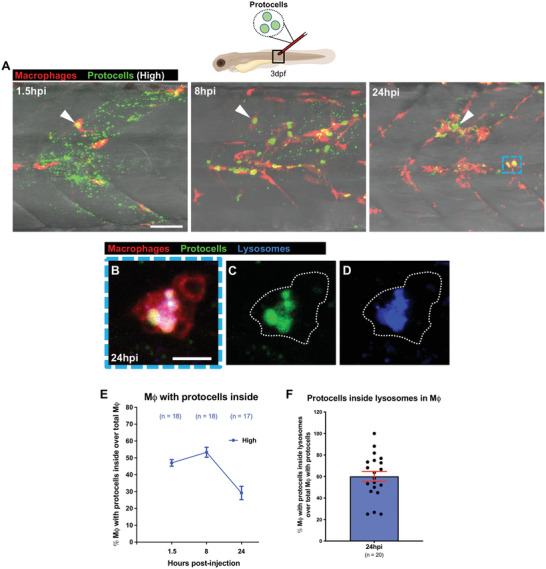
Locally injected protocells are taken up by macrophages. A) Multi‐channel confocal images of the flank of Tg(*mpeg1:mCherry*) larvae after local injection of FITC‐protocells at 3 dpf and imaged at 1.5, 8, and 24 hpi showing the distribution of protocells within macrophages (white arrowheads) and dispersed along the fish somite (injection site). See also Movie S9, Supporting Information. B–D) Multi‐channel (B) or single‐channel (C,D) confocal images showing the lysosomal fate of internalized FITC‐protocells within a macrophage (white dotted outlines) after local injection of protocells at 24 hpi (blue dashed box in [A]). E,F) Graphs showing percentage of macrophages containing protocell(s) (E) or with overlaying protocells and lysosomes (F) quantified from the regions imaged in (A). “High” corresponds to the protocell concentration injected (1.25 × 10^7^ protocells/µL). Accompanying schematic illustrates developmental stage (larva), type of injection (local), and imaged area (black outlined box) used for the experiment. Data are pooled from three independent experiments. Graphs show mean ± SEM. In graph (E) each dot represents the mean of all fish analyzed, and in graph (F) each dot represents one fish. Mϕ = macrophages; *n* = number of fish. Scale bars = 50 µm (A), 10 µm (B).

To test whether local PC injection might also be a therapeutic strategy for loading tissue‐resident macrophages, we injected a high concentration of dextran‐containing PCs without anti‐miR223 cargo into a single somite on the flank of 3 dpf Tg(*mpeg1:mCherry*) larvae, and by 1.5 hpi we saw that almost 50% of all macrophages in the injected region had taken up the proteinosomes, reaching values of above 50% at 8 hpi and reducing to 30% by 24 hpi (**Figure** **3**A,E and Movie S9, Supporting Information). Co‐staining of these flank PC‐loaded macrophages with Lysotracker, an intravital lysosomal dye, at 24 hpi, indicates that phagocytosed PCs appear to be processed through lysosomes (Figure 3B–D,F), in line with previous zebrafish studies of lysosomal targeting of lipid‐based particles in macrophages.^[^ ^42^ ^]^

### 2.3 In Vivo Uptake of Anti‐miR223 PCs Triggers Enhanced Pro‐Inflammatory State in Macrophages

**Figure 4:**
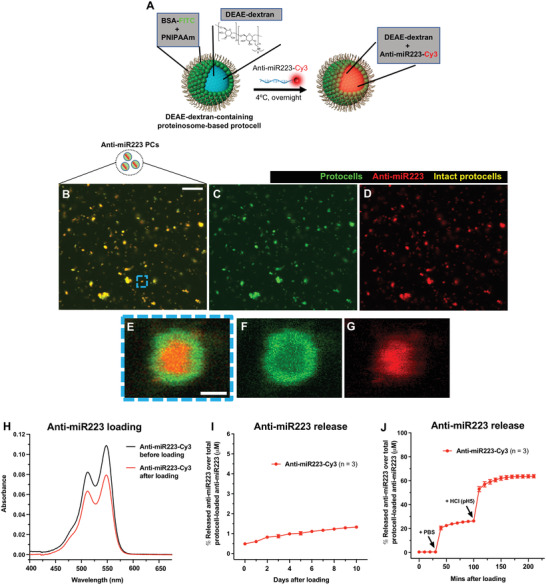
Loading strategy of anti‐miR223 into protocells. A) Schematic of the experimental design for loading DEAE‐dextran‐containing FITC‐protocells with anti‐miR223‐Cy3. B–G) Multi‐channel (B,E) or single‐channel (C,D,F,G) confocal images of FITC‐protocells after loading with anti‐miR223‐Cy3. E–G) High magnification views of (B)–(D) showing a single anti‐miR223‐Cy3 FITC‐protocell. H) Spectra showing absorbance quantification of the anti‐miR223‐Cy3 supernatant before and after protocell loading. I,J) Graphs showing percentage of anti‐miR223‐Cy3 released from loaded protocells into the supernatant over total anti‐miR223‐Cy3 concentration initially loaded into protocells, under normal conditions in H_2_O (I) or after exposure to PBS and HCl (J). Data are representative (H) or pooled (I,J) from three independent experiments. Graphs (I,J) show mean ± SEM, and each dot represents the mean of all experiments analyzed. *n* = number of experiments; PCs = protocells. Scale bars = 20 µm (B), 1 µm (E).

**Figure 5:**
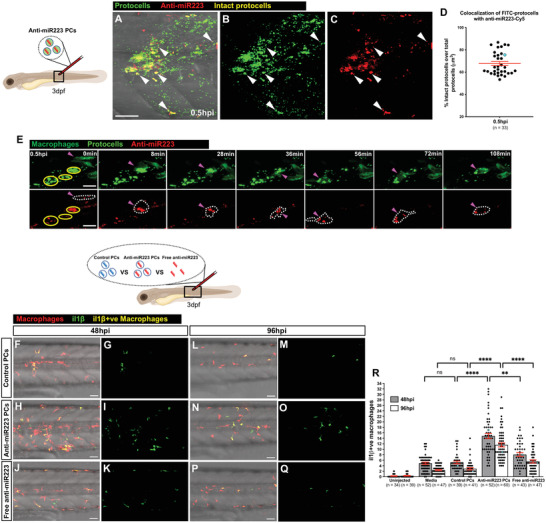
Uptake of anti‐miR223 protocells enhances il1*β* expression in macrophages. A–C) Multi‐channel (A) or single‐channel (B,C) confocal images of the flank of a 3 dpf casper larva after local injection of anti‐miR223‐Cy5 FITC‐protocells at 0.5 hpi; white arrowheads indicate anti‐miR223 protocells that remain intact post injection. D) Dot plot showing percentage of intact protocells as quantified by colocalization of FITC (protocells) with Cy5 (anti‐miR223) from the regions imaged in (A)–(C). E) Single‐channel confocal movie frames of a region of the flank of a 3 dpf Tg(*mpeg1:FRET*) larva after local injection of anti‐miR223‐Cy5 FITC‐protocells at 0.5 hpi showing the uptake of intact protocells (yellow circles) by a macrophage (magenta arrowheads and white dotted outlines). See also Movie S10, Supporting Information. F–Q) Multi‐channel (F,H,J,L,N,P) or single‐channel (G,I,K,M,O,Q) confocal images of the flank of Tg(*mpeg1:mCherry*;*il1β:GFP*) larvae showing il1*β*‐positive macrophages (yellow) (il1*β*‐negative macrophages are red) after local injection of unlabeled control protocells, unlabeled anti‐miR223 protocells or unlabeled free anti‐miR223 at 3 dpf and imaged at 48 hpi (F–K) and 96 hpi (L–Q). R) Graph showing the number of il1*β*‐positive macrophages following each treatment quantified from the regions imaged in (F)–(Q). See also Figures S7 and S8, Supporting Information. Accompanying schematics illustrate developmental stage (larva), type of injection (local), and imaged area (black outlined box) used for each experiment. Data are pooled from three independent experiments and analyzed using Kruskal–Wallis test with Dunn's multiple comparisons test (R), ns *p* ≥ 0.05, **p < 0.01, ****p < 0.0001. Graphs show mean ± SEM, each dot represents one fish and blue dots correspond to the representative images shown in the panels. *n* = number of fish; PCs = protocells. Scale bars = 50 µm (A,F,H,J,L,N,P), 20 µm (E).

Having established that PCs can be taken up by leukocytes in vivo, we next tested whether the PCs might effectively deliver anti‐miR223 to these leukocytes to transiently recapitulate the miR223 knockout/knockdown, anti‐cancer phenotype we described above. We first loaded the proteinosomes with anti‐miR223 by electrostatically induced complexation with pre‐encapsulated DEAE‐dextran (**Figure** **4**A). Fluorescent labeling of the 3′ end of anti‐miR223 allows us to track cargo retention within fluorescein isothiocyanate (FITC)‐PCs (Figure 4B–G). Absorbance analyses suggest that anti‐miR223 is loaded into DEAE‐dextran‐containing PCs at a local concentration of about 125 µm (12.5 µm in bulk solution containing 3.2 × 10^7^ PCs/µL) (Figure 4H), and that there is almost no leakage from the PCs into the surrounding media up to 10 days after initial loading (Figure 4I). Significantly, complexation between DEAE‐dextran and anti‐miR223 is reversible such that free anti‐miR223 is released by increasing the ionic strength or decreasing the pH to values observed in lysosomes (pH 5) (Figure 4J). We then used the DEAE‐dextran‐containing FITC‐PCs loaded with fluorescently labeled anti‐miR223 in vivo and confirmed that up to 70% of PCs remain structurally intact, retaining their anti‐miR223 cargoes, over a period of 0.5 h post local injection in 3 dpf casper larvae (**Figure** **5**A–D). Furthermore, the anti‐miR223‐loaded PCs are effectively taken up by recruited zebrafish macrophages in 3 dpf Tg(*mpeg1:FRET*) larvae (Figure 5E and Movie S10, Supporting Information).

To determine whether injection of PCs loaded with anti‐miR223 is able to reprogram macrophages in vivo to make them more pro‐inflammatory, and whether this is a more effective strategy than delivery of free anti‐miR223, we locally injected anti‐miR223 PCs, control PCs (loaded with scrambled anti‐miR), or free anti‐miR223, into the somites of a zebrafish interleukin 1*β* (il1*β*) reporter line (combined with Tg(*mpeg1:mCherry*)), which reveals expression levels of this cytokine which is classically associated with a pro‐inflammatory phenotype.^[^ ^43^ ^]^ The results indicate considerably higher levels of il1*β*‐positive macrophages in tissues treated with anti‐miR223‐loaded PCs at 48 hpi, and this pro‐inflammatory state appears more persistent than in a control‐loaded PC group extending up until 96 hpi (Figure 5F–I,L–O,R). Injection of the control PCs did not result in increased numbers of il1*β*‐positive macrophages compared to media‐injected fish at either 48 or 96 hpi, suggesting that the proteinosomes in themselves do not trigger a pro‐inflammatory state (Figure 5R). Injection of free anti‐miR223 results in a small increase in il1*β* upregulation compared with fish injected with media or control‐loaded PCs, but this upregulation is considerably lower than in those injected with anti‐miR223 PCs (Figure 5F–R and Figure S7, Supporting Information). These observations correlate with the low uptake of fluorescently labeled free anti‐miR223 by macrophages compared with when delivered in PCs (Figure S8, Supporting Information). Neutrophils, whilst less efficient at uptake of PCs, also appear to be reprogrammed to the extent that they also express higher levels of il1*β* after local injection of PCs carrying anti‐miR223 cargo (Figure S9, Supporting Information).

### 2.4 PCs Effectively Deliver Anti‐miR223 to Reprogram Human Macrophages In Vitro

**Figure 6:**
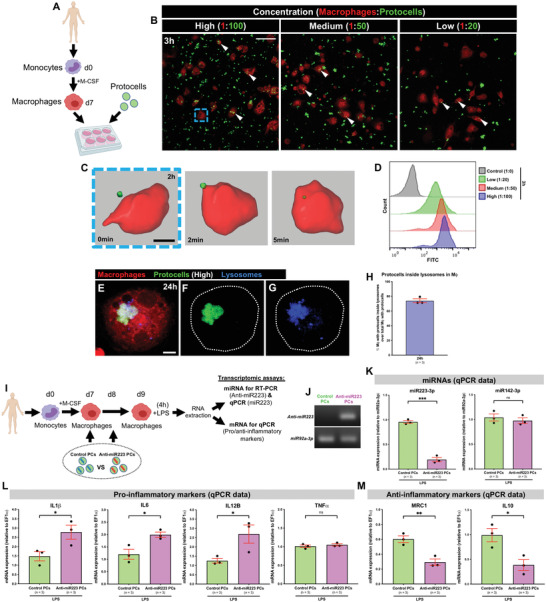
In vitro uptake of anti‐miR223 protocells by human macrophages enhances expression of pro‐inflammatory markers. A) Schematic of the experimental design; monocytes isolated from human donors undergo in vitro differentiation toward macrophages (via M‐CSF) prior to FITC‐protocell addition (day 7). B) Multi‐channel confocal images of human macrophages after incubation with different FITC‐protocell concentrations for 3 h; white arrowheads indicate protocells within macrophages. C) Imaris 3D reconstruction from confocal movie frames showing FITC‐protocell uptake by a human macrophage 2 h after protocell addition (blue dashed box in [B]). See also Movie S11, Supporting Information. D) Representative histograms from flow cytometry analysis of human macrophages treated with different FITC‐protocell concentrations for 3 h. See also Figure S10, Supporting Information. E–G) Multi‐channel (E) or single‐channel (F,G) confocal images showing the lysosomal fate of internalized FITC‐protocells within a human macrophage (white dotted outlines) 24 h after protocell addition. H) Graph showing percentage of human macrophages with overlaying protocells and lysosomes 24 h after protocell addition quantified from the regions imaged in (B). I) Schematic of the experimental timeline to evaluate human macrophage reprogramming and anti‐miR223 delivery/function (through transcriptomic analysis) after anti‐miR223 protocell treatment; FITC‐protocells, loaded with control anti‐miR or anti‐miR223, were administered to macrophage cultures in two consecutive doses (days 7 and 8) prior to LPS exposure for 4 h (day 9), and total RNA from macrophages was extracted for RT‐PCR and qPCR assays. J) RT‐PCR of miRNA extracted from human macrophages after each protocell treatment; anti‐miR223 from macrophages treated with control protocells was used as a negative control, and miR92a‐3p from macrophages of each protocell treatment as a loading control. K–M) Graphs showing qPCR data for the expression levels of miRNAs (miR223‐3p and miR142‐3p) (K), pro‐inflammatory markers (IL1*β*, IL6, IL12B, and TNF*α*) (L), and anti‐inflammatory markers (MRC1 and IL10) (M), in LPS‐stimulated human macrophages after each protocell treatment. See also Figures S11 and S12, Supporting Information. qPCR data were normalized to the indicated housekeeping genes/miRs from LPS‐stimulated macrophages that had not been treated with protocells. “High”, “medium”, and “low” correspond to the different macrophage:protocell ratios used (1:100, 1:50, and 1:20, respectively). Data are from one experiment (J), pooled from three independent experiments (H,K–M), or representative from two independent experiments (D), and analyzed using unpaired two‐sided *t*‐test, ns *p* ≥ 0.05, *p < 0.05, **p < 0.01, ***p < 0.001. Graphs show mean ± SEM, and each dot represents one experiment. Mϕ = macrophages; *n* = number of experiments; PCs = protocells. Scale bars = 50 µm (B), 10 µm (C), 5 µm (E).

To more fully investigate the feasibility of using PCs to transiently deliver “reprogramming” anti‐miR223 cargoes to macrophages, and whether this might extend to human immune cells, we established an in vitro assay with primary human macrophages differentiated from peripheral blood monocytes isolated from human donors and subsequently treated with macrophage colony‐stimulating factor (M‐CSF) (**Figure** **6**A). To evaluate the potential for PC uptake, DEAE‐dextran‐containing proteinosomes, initially without anti‐miR223 cargo, were added to the macrophage culture, and as with the in vivo zebrafish studies, human macrophages rapidly phagocytosed the PCs (Figure 6B–D). More than 80% of the macrophages contained one or more PCs by 3 h of media supplementation with high PC concentrations (macrophage:PC ratio = 1:100), as assayed by confocal microscopy and by flow cytometry analysis (Figure 6B,D and Figure S10A,B, Supporting Information). Uptake of PCs occurs at a slightly slower pace than in the zebrafish model, taking 3 min from surface contact to complete internalization (Figure 6C and Movie S11, Supporting Information). But, as with the zebrafish studies, internalized PCs are targeted to intracellular lysosomes in human macrophages as shown by Lysotracker staining 24 h following supplementation with a high concentration of PCs (Figure 6E–H).

We exploited the combination of high anti‐miR223 loading and triggerable cargo release along with effective cellular uptake properties to reprogram human macrophages in vitro. Flow cytometry analysis shows that anti‐miR223‐containing PCs are taken up by a similar percentage of human macrophages as observed for anti‐miR223‐free PCs 3 h after PC addition (Figure S10, Supporting Information). To address whether intracellular release of anti‐miR223 from phagocytosed PCs can reprogram human macrophages toward a more persistent pro‐inflammatory (and thus potentially anti‐cancer) phenotype, we performed transcriptional analysis of human macrophages after addition of PCs accompanied by transient lipopolysaccharide (LPS) exposure to initiate the pro‐inflammatory phenotype switch^[^ ^44^, ^45^ ^]^ (Figure 6I). Using this experimental design, we detect anti‐miR223 levels, as revealed by reverse transcription polymerase chain reaction (RT‐PCR), only in those macrophages exposed to anti‐miR223 PCs, whereas anti‐miR223 was undetectable in macrophages alone (without PCs) or those incubated with PCs loaded with a control scrambled anti‐miR (Figure 6J), confirming that proteinosomes can effectively deliver anti‐miR223 to human macrophages in vitro. Additionally, quantitative polymerase chain reaction (qPCR) analysis showed that macrophages incubated with anti‐miR223 PCs exhibit a more than fourfold reduction in miR223 (miR223‐3p) expression by comparison to control PCs, but identical levels of miR142‐3p, a control miR also expressed by macrophages (Figure 6K), indicating that this approach is effective and specific for miR223 inhibition. As a consequence of miR223 depletion via PCs, we observe an increase of almost 50% in mRNA levels of the pro‐inflammatory cytokine IL1*β* in the anti‐miR223 PC‐treated macrophages (Figure 6L). These macrophages also exhibit an upregulation of other known pro‐inflammatory cytokines, including interleukin 6 (IL6) and interleukin 12B (IL12B), but no change in tumor necrosis factor *α* (TNF*α*) (Figure 6L), in agreement with previous murine macrophage studies.^[^ ^46^ ^]^ Anti‐inflammatory markers, mannose receptor c‐type 1 (MRC1) and interleukin 10 (IL10), are downregulated compared to control PC‐treated macrophages (Figure 6M), further supporting that anti‐miR223 PC treatment enhances the pro‐inflammatory phenotype. No differences in the expression of these pro/anti‐inflammatory cytokines were observed when anti‐miR223 PCs were added to cultured macrophages in the absence of LPS (Figure S11, Supporting Information).

To confirm that these transcriptional changes in cytokine mRNA levels, triggered by anti‐miR223 PC reprogramming, reflect altered secretion of key cytokines, we performed Luminex‐based multiplex immunoassays which indeed showed modest, but significant, increases in IL1*β*, IL6, and IL12B, and downregulation of IL10 protein levels (Figure S12A–C, Supporting Information). TNF*α* protein levels remained the same (Figure S12B, Supporting Information), reflecting our transcriptomic data. Levels of secreted IL1*β* by human macrophages were also quantified by an enzyme‐linked immunosorbent spot (ELISpot) assay, which indicated a clear increase in number of IL1*β*‐secreting cells and levels of IL1*β* secretion by these cells after anti‐miR223 PC treatment (Figure S12A,D,E, Supporting Information).

### 2.5 Anti‐miR223 PC‐Mediated Reduced Cancer Growth in Zebrafish

The above studies in non‐cancerous larvae and human macrophages indicate that it may be possible to replicate the genetic reprogramming of leukocytes via loading with anti‐miR223 PCs to change phenotype in ways that might make them better able to suppress cancer cell proliferation. To test this, we locally injected anti‐miR223 PCs, singly or on three consecutive days, into the same somite of 3 dpf Ras‐expressing larvae (Tg(*kita:HRAS^G12V^‐GFP*)), which have a predisposition to develop pre‐neoplastic growths. A single injection of anti‐miR223 PCs did not alter pre‐neoplastic cell numbers (Figure S13A–D,I, Supporting Information), but three injections over the course of 3 days lead to a significantly reduced number of pre‐neoplastic cells in that region of the flank by comparison to injection of control scrambled‐anti‐miR‐loaded PCs (Figure S13E–H,J, Supporting Information). These results are consistent with previous studies of zebrafish larvae, which indicated that reprogramming inflammation by infection tended to only block cancer growth if the stimuli were sustained.^[^ ^11^ ^]^

**Figure 7:**
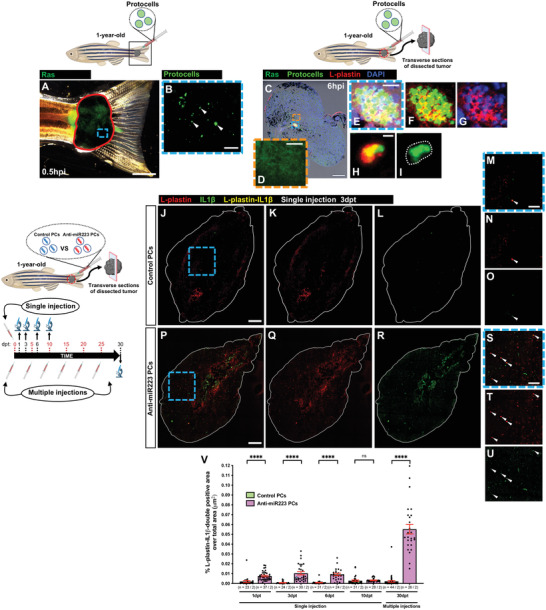
Uptake of anti‐miR223 protocells induces a leukocyte pro‐inflammatory state in adult zebrafish. A) Multi‐channel image of a 1‐year‐old adult tail tumor (red outline) locally injected with FITC‐protocells and imaged at 0.5 hpi. B) High magnification view of (A) showing a single‐channel image of FITC‐protocells (white arrowheads) at the injection site. C) Multi‐channel confocal image of an immunostained cryosection from a 1‐year‐old adult tail tumor harvested 6 h after local FITC‐protocell injection; nuclei are stained with DAPI (blue), and leukocytes are revealed by anti‐L‐plastin immunostaining (red). D) High magnification view of (C) showing single‐channel confocal image of Ras‐GFP region. E–I) High magnification views of (C) showing multi‐channel (E–H) or single‐channel (I) confocal images of FITC‐protocells within L‐plastin‐positive cells (white dotted outline in [I]). See also Figure S14, Supporting Information. J–U) Multi‐channel (J,M,P,S) or single‐channel (K,L,N,O,Q,R,T,U) confocal images of immunostained cryosections from 1‐year‐old adult tail tumors at 3 dpt after a single local injection of unlabeled control protocells (J–O) or unlabeled anti‐miR223 protocells (P–U); white lines indicate tumor margins; leukocytes are revealed by anti‐L‐plastin immunostaining (red) and IL1*β* revealed by anti‐IL1*β* immunostaining (green). (M–O,S–U) High magnification views of (J)–(L) and (P)–(R); white arrowheads indicate L‐plastin‐IL1*β*‐double‐positive cells (yellow). V) Graph showing percentage of L‐plastin‐IL1*β*‐double‐positive area over total tumor area from sections after each injection regime quantified from the regions imaged in (J)–(L) and (P)–(R). Accompanying schematics illustrate fish age, timeline, type of injection (local, single or multiple), and imaged area (black outlined box) used for each experiment. Data are pooled from two independent experiments, and analyzed using Kruskal–Wallis test with Dunn's multiple comparisons test, ns *p* ≥ 0.05, ****p < 0.0001. Graph shows mean ± SEM, each dot represents one section and blue dots correspond to the representative images shown in the panels. *n* = number of sections/fish; PCs = protocells. Scale bars = 2 mm (A), 500 µm (B), 200 µm (C), 25 µm (D,E), 5 µm (H), 300 µm (J,P), 100 µm (M,S).

To investigate whether this approach might be upscaled as a feasible therapeutic strategy for dissolving larger, more established, and growing cancers, we repeated the above experiments in Ras‐expressing adult fish (Tg(*kita:HRAS^G12V^‐GFP*)). While the opaque tissues of an adult fish melanoma are much less amenable to live imaging than the translucent larval tissues described above, it is still apparent that injected DEAE‐dextran‐containing fluorescently tagged PCs (without anti‐miR223) remain close to the injection site in the melanoma at 0.5 hpi (**Figure** **7**A,B). Sections through the cancerous tissue of these injected tailfin melanomas at 6 hpi show fluorescent PCs encapsulated within clusters of L‐plastin‐positive immune cells, suggesting that, consistent with the larval studies, there is a rapid local uptake of PCs by leukocytes (Figure 7C–I). This is not the case for free anti‐miR223 which is diluted by rapid diffusion from the injection site, leading to occasional detection of some anti‐miR223 levels at the tumor border, and appears not to be taken up by immune cells (or cancer cells) in the vicinity (Figure S14, Supporting Information).

To determine whether anti‐miR223 PCs could change the phenotype of leukocytes in the tumor within the vicinity of the injection area, we injected tailfin melanomas of Ras‐expressing adult fish (Tg(*kita:HRAS^G12V^‐GFP*)) with PCs loaded with either scrambled‐anti‐miR (control) or anti‐miR223 cargo, dissected the tumor, and immunostained sections of these tumors with anti‐L‐plastin and anti‐IL1*β* antibodies at several timepoints post injection. Our data show that a single injection of anti‐miR223 PCs effectively reprograms leukocytes at the injected tumor site, leading to a transient high pro‐inflammatory phenotype, as revealed by anti‐IL1*β* immunostaining, extending for up to 6 days post treatment (dpt), but this diminished to control levels at later timepoints (10 dpt) (Figure 7J–V). When tumors received repetitive injections of anti‐miR223 PCs, leukocytes retain their pro‐inflammatory profile over a considerable time (Figure 7V), suggesting that this approach might potentially be a more effective anti‐cancer therapy compared with the single dose strategy.

**Figure 8:**
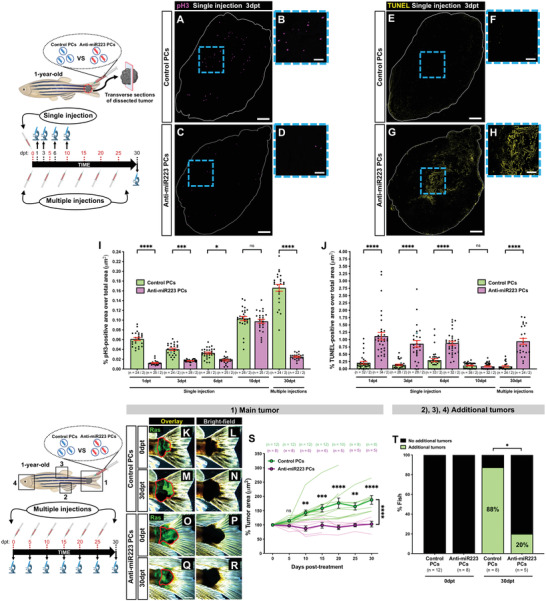
Anti‐miR223 protocell treatment reduces cancer progression in adult zebrafish. A–H) Single‐channel confocal images of immunostained cryosections from 1‐year‐old adult tail tumors at 3 dpt after a single local injection of unlabeled control protocells (A,B,E,F) or unlabeled anti‐miR223 protocells (C,D,G,H); white lines indicate tumor margins; proliferating cells are revealed by anti‐pH3 immunostaining (magenta) in (A)–(D) and apoptotic cells revealed by TUNEL staining (yellow) in (E)–(H). B,D,F,H) High magnification views of (A), (C), (E), and (G). I,J) Graphs showing percentage of pH3‐positive (I) or TUNEL‐positive (J) area over total tumor area from sections after each injection regime quantified from the regions imaged in (A), (C), (E), and (G). K–R) Multi‐channel (K,M,O,Q) or single‐channel (L,N,P,R) images of 1‐year‐old adult tail tumors (red outlines) at 0 and 30 dpt after multiple local injections of unlabeled control protocells (K–N) or unlabeled anti‐miR223 protocells (O–R). S) Graph showing adult tail tumor growth curves after each protocell treatment quantified from the regions imaged in (K)‐(R) and (1). See also Figure S15, Supporting Information. T) Bar chart showing percentage of fish bearing tailfin tumors, and any additional tumor (or not), after each protocell treatment at 0 and 30 dpt quantified from the regions imaged in (2)–(4). Accompanying schematics illustrate fish age, timeline, type of injection (local, single or multiple), and imaged area (black outlined box) used for each experiment. Data are pooled from two independent experiments, and analyzed using Kruskal–Wallis test with Dunn's multiple comparisons test (I,J), two‐way ANOVA test with Bonferroni's multiple comparisons test (S), or Fisher's exact test (T), ns *p* ≥ 0.05, *p < 0.05, **p < 0.01, ***p < 0.001, ****p < 0.0001. Graphs (I), (J), and (S) show mean ± SEM. In graphs (I) and (J), each dot represents one section and blue dots correspond to the representative images shown in the panels. In graph (S), each dot represents the mean of all fish analyzed and curves in lighter color correspond to individual fish. *n* = number of sections/fish (I,J); *n* = number of fish (S,T); PCs = protocells. Scale bars = 300 µm (A,C,E,G), 100 µm (B,D,F,H), 2 mm (K,M,O,Q).

To address whether proliferation of cancer cells within the injected melanoma is altered by the presence of reprogrammed leukocytes, we stained sections of cancerous tissue with antibodies against phospho‐histone H3 (pH3) after injection of PCs at different timepoints. From 1 to 6 dpt, the cancerous tissue exposed to a single dose of anti‐miR223 PCs exhibited a significantly reduced local proliferative index by comparison to tumor sites where control PCs had been injected; but this reduction in proliferation ceased by 10 dpt (**Figure** **8**A–D,I). However, multiple injections of anti‐miR223 PCs maintain lower cell proliferation rates compared to control until at least 30 dpt, giving the most dramatic reduction at this timepoint (Figure 8I).

The extent of cell death within cancerous tissue near to the injection site was investigated by measuring the levels of apoptosis by terminal deoxynucleotidyl transferase dUTP nick end labeling (TUNEL) staining. A significant difference between control and anti‐miR223‐loaded PCs was observed with the latter driving increased levels of apoptosis after a single injection at 1, 3, and 6 dpt, and after multiple injections at 30 dpt (Figure 8E–H,J).

Finally, to determine if reprogramming of innate immune cells alters cancer progression, we performed multiple injections of anti‐miR223 PCs in tailfin melanomas of 1‐year‐old Ras‐expressing adult fish (Tg(*kita:HRAS^G12V^‐GFP*)), and measured the tumor area/volume in comparison with melanomas injected with control PCs (loaded with scrambled anti‐miR). Our results indicate that whilst control PC‐treated tailfin tumors continued growing over time, in fish where leukocytes had been locally reprogrammed with anti‐miR223 PCs, their tumors remained stable or decreased in size (Figure 8K–S and Figure S15A–J, Supporting Information). As depigmentation seems to be a common feature of melanoma regressions in the clinic,^[^ ^47^ ^]^ we monitored pigmentation in the tailfin tumors and observed that tumors treated with anti‐miR223 PCs had reduced overall pigmentation at 30 dpt (Figure S15K–O, Supporting Information). In addition, the percentage of fish growing additional tumors at distant sites from their tailfin melanomas (i.e., in head, dorsal, or anal fins) was decreased after treatment with anti‐miR223 PCs (Figure 8T). This suggests that the anti‐cancer effects of these local injections might even extend beyond the injection site, leading to a partial systemic or abscopal effect.

## 3 Discussion

Our proof of principle studies indicate that therapeutic reprogramming of leukocytes in vivo can significantly inhibit cancer progression in a zebrafish model of melanoma. We first show that the miR223, which often associates with poor cancer prognosis in patients,^[^ ^13^, ^14^, ^15^, ^16^, ^17^ ^]^ is a good target with a series of genetic knockout and knockdown experiments that result in reduced levels of cancer in juvenile and adult fish. We then test a novel therapeutic strategy involving targeted knockdown of miR223 in neutrophils and macrophages by PC delivery of anti‐miR223 to these phagocytic lineages, and show successful leukocyte reprogramming leading to growth suppression of established adult melanomas. To our knowledge, this leukocyte reprogramming is the first in vivo demonstration of a functional application for non‐lipid PCs in cancer immunotherapy.

Previous studies have reported how liposomes injected into the vasculature of zebrafish tend to accumulate in non‐specific tissues (e.g., endothelium) and have rather short circulation times, making them less optimal for sustained leukocyte reprogramming and cancer treatments.^[^ ^48^ ^]^ Here we demonstrate how the use of proteinosome‐based PCs can overcome these delivery issues in vivo, as they remain stable and have extensive circulation times, both attributes beneficial for potential prolonged therapeutic effects of anti‐miR223‐loaded PCs. In addition, we chose proteinosome‐based PCs over other potential delivery vectors because they enable encapsulation and delivery of high concentrations of the reprogramming cargo and offer the potential for bespoke surface engineering^[^ ^49^, ^50^ ^]^ and endogenous information processing.^[^ ^25^, ^51^ ^]^ Consequently, while we demonstrate that local injection of PCs and reprogramming of leukocytes at a primary cancer site can lead to abscopal suppression of cancers distant to this injection site, systemic IV injection of PCs with the capacity to home to all cancer sites, by engineered PC‐surface receptors, would clearly enhance the potential of this therapy to specifically target multiple, invasive cancers.

Will these strategies be appropriate for treating human cancers? Our experiments in zebrafish are early pre‐clinical studies, but we also show that human macrophages, at least in vitro, can be similarly reprogrammed. As miR223 has an established role in regulating the pro‐inflammatory phenotype of leukocytes, and is very well conserved across species,^[^ ^20^ ^]^ it may be a therapeutic target, not just for melanoma but for other solid tumors where it also appears to be linked to poor patient prognosis.^[^ ^14^, ^17^ ^]^ Clearly, such a treatment would be less appropriate for tumor types where there is no demonstrated association with miR223 expression levels.

Moreover, other known anti‐inflammatory miRs could also be similarly targeted by PC‐delivered phagocytic uptake, for example, miR21,^[^ ^52^ ^]^ miR132,^[^ ^53^ ^]^ or miR146.^[^ ^54^ ^]^ Alternatively, our novel PC vector approach could be adapted for delivery of small molecules for immune cell reprogramming such as synthetic agonists of endosomal TLR7 (Imiquimod and 852A), TLR7/8 (Resiquimod and 3M‐052), and TLR9 (IMO‐2055), which have previously been shown to effectively re‐polarize macrophages in ways that enhance their anti‐tumoral activities in mouse models, and one of which, Imiquimod, is currently approved for local treatment of superficial basal cell carcinoma.^[^ ^55^, ^56^, ^57^ ^]^ Taken together, the PC‐based delivery of leukocyte reprogramming cargoes could offer a novel strategy for precise targeting of otherwise hard‐to‐access cancers that might be vulnerable to sustained pro‐inflammatory activity, perhaps in synergy with other immune‐mediated cancer therapeutics, or as an adjuvant to cancer radiotherapy.

## 4 Experimental Section

### Preparation of Proteinosome‐Based PCs

BSA‐NH_2_ was synthesized by carbodiimide‐activated conjugation of 1,6‐diaminohexane (Sigma) to aspartic and glutamic acid residues on the external surface of the protein. The coupling reaction was initiated by the immediate addition of *N*‐ethyl‐*N*′‐(3‐(dimethylaminopropyl)carbodiimide hydrochloride (Sigma). pH was maintained at 6.5, and the solution was stirred (6 h) prior to centrifugation and dialysis against Milli‐Q H_2_O. The final product was obtained by freeze‐drying.

End‐capped mercaptothiazoline‐activated PNIPAAm, synthesized as previously described,^[^ ^23^ ^]^ was added to a solution of BSA‐NH_2_. The mixed solution was stirred (12 h) and then purified using a centrifugal filter (50 kDa) to remove unreacted PNIPAAm and salts. The BSA‐NH_2_/PNIPAAm conjugate was obtained by freeze‐drying.

PCs were prepared by mixing an aqueous BSA‐NH_2_/PNIPAAm solution with 2‐ethyl‐1‐hexanol (Sigma) followed by sonication (5 min, 300 W). Typically, aqueous BSA‐NH_2_/PNIPAAm (pH 8.5, 20 µL, 30 mg mL^−1^) was mixed with polymers (dextran/DEAE‐dextran, Sigma, 150 kDa, pH 8.5, 10 µL, 10 mg mL^−1^) and *O*,*O*′‐bis[2‐(*N*‐succinimidyl‐succinylamino)ethyl]polyethylene glycol (Sigma, pH 8.5, 5 µL, 0.4 mg µL^−1^, cross‐linker). After mixing, 2‐ethyl‐1‐hexanol (750 µL) was added to the solution, followed by sonication (5 min, 300 W).

Transfer of the cross‐linked PCs into H_2_O was achieved by collecting the upper oil layer 48 h post sedimentation, and then centrifugation (10 000 rpm, 10 min) to obtain ultra‐small proteinosomes (2–5 µm). Suspensions were washed (70% ethanol [3×], H_2_O [1×]), dispersed in H_2_O, and preserved at 4 °C.

For preparation of FITC‐PCs, PNIPAAm and FITC‐labeled BSA‐NH_2_ were mixed to obtain BSA‐NH_2_‐FITC/PNIPAAm conjugate. In brief, FITC (Sigma) was added to BSA‐NH_2_/PNIPAAm (pH 8.5) and the solution was stirred at 4 °C (overnight), purified by dialyzing against Milli‐Q H_2_O and freeze‐dried.

FITC‐PC numbers (1:400, 1:800, 1:1600, and 1:3200 dilutions) were quantified by flow cytometry using an Acea NovoCyte Flow Cytometer. Unlabeled PCs were prepared in parallel with FITC‐PCs using the protocol described above and the number of both PC types was assumed to be equivalent.

### Anti‐miR223 Loading into PCs

For anti‐miR loading into PCs, DEAE‐dextran‐containing PCs were incubated with the anti‐miR (Zebrafish‐anti‐miR223: 5′‐GGGGUAUUUGACAAACUGACA‐3′ [Mw ≈ 6.7 kDa]; Human‐anti‐miR223: 5′‐UGGGGUAUUUGACAAACUGACA‐3′ [Mw ≈ 7.2 kDa]) solution (50 µm) at 4 °C (overnight) and washed to remove unloaded anti‐miRs. The anti‐miR loading efficiencies were monitored by UV/Vis absorption spectra. PCs were imaged with a Leica SP5‐II AOBS confocal laser scanning microscope using 63× oil lens. Images were processed using Fiji and displayed as maximum projections. Unlabeled control anti‐miR (4464076) and unlabeled anti‐miR223 (4464084) were purchased from ThermoFisher, and anti‐miR223‐cyanine 3/cyanine 5 (Cy3/Cy5) from Integrated DNA Technologies. Zebrafish‐anti‐miR223 was used for all in vivo fish experiments, and Human‐anti‐miR223 for the in vitro human macrophage studies.

### PBMNC and CD14^+^ Isolation

Peripheral blood mononuclear cells (PBMNCs) were isolated from platelet apheresis blood waste (NHSBT, Bristol, UK) from anonymous healthy donors. PBMNC separation was performed using PBMNC Spin Medium (pluriSelect Life Science) as previously described.^[^ ^58^, ^59^ ^]^ Briefly, blood from apheresis cones was diluted 1:1 with Hank's Balanced Salt Solution (HBSS, Sigma) containing 0.6% acid citrate dextrose and layered over PBMNC spin medium. Samples were centrifuged to generate a density gradient and PBMNCs collected from the interface. CD14^+^ cells were isolated from PBMNCs using a magnetic micro‐bead CD14^+^ kit (Miltenyi Biotec), LS columns (Miltenyi Biotec), and MidiMACS separators (Miltenyi Biotec) as previously described.^[^ ^60^ ^]^ CD14^+^ cells were stored frozen in 50% fetal bovine serum (FBS, Gibco) and 40% PBS with 10% DMSO (Sigma) in liquid nitrogen until required.

### Human Macrophage Ex Vivo Culture and PC Administration

Thawed cells were washed and resuspended at a density of 0.17 × 10^6^–0.33 × 10^6^ cells/mL in RPMI 1640 (Gibco) supplemented with 10% FBS, 25 ng mL^−1^ M‐CSF (Miltenyi Biotec), and penicillin/streptomycin (Sigma) at 100/0.1 U mg^−1^ mL^−1^ of media, respectively, with/without the inclusion of 100 ng mL^−1^ LPS (Sigma) for 4 or 8 h prior to harvesting. Cells were incubated at 37 °C with 5% CO_2_. Cells were harvested from the adherent macrophage culture via scraping and using a detaching buffer (10 mm EDTA and 15 mm Lidocaine in PBS) where required. 4 × 10^5^, 1 × 10^5^, or 2.6 × 10^5^ macrophages were used for transcriptomic/immunoassay, flow cytometry, or imaging analyses, respectively, per treatment for each experiment. Macrophages from different donors were used for each experiment. PCs were sterilized by gamma‐radiation (40 Gy) prior to anti‐miR loading and/or incubation with macrophages. In all in vitro experiments, PCs were administered to macrophage cultures in a high concentration (macrophage:PC ratio = 1:100), unless otherwise stated, and in a single dose (day 7), or for transcriptomics/immunoassays, in multiple doses (days 7 and 8).

### Flow Cytometry Analysis of PC Uptake by Human Macrophages

Analysis of PC uptake by primary human macrophages was conducted using flow cytometry 3 h after PC supplementation (day 7). Cells were harvested as above, washed, and immediately analyzed for presence of FITC‐PCs. Gating strategy was established as follows: a cell gate was first applied using the FSC and SSC to gate out free PCs and debris, and the macrophage only and PC only controls were then used to create a loaded macrophage gate based on FITC signal whilst excluding aggregated PCs found within the cell gate. Data were collected using a MACSQuant Flow Cytometer and analyzed using FlowJo (Version 10.7).

### Luminex‐Based Multiplex Immunoassay for Analysis of Cytokine Secretion by Human Macrophages

Supernatant cytokine levels for human IL1*β*, IL6, IL12B, TNF*α*, and IL10 were measured using a Human Procartaplex Mix & Match 5‐plex immunoassay kit (ThermoFisher) following manufacturer's instructions. Supernatants from cultured human macrophages were collected in a tube and centrifuged (14 000 rpm, 10 min) at 4 °C to remove particulates, and the clarified medium was transferred into a clean tube for immediate use. Undiluted samples were added to a 96‐well plate that contained the washed capture antibody‐conjugated magnetic beads, and the plate was shaken (600 rpm, 30 min) at room temperature prior to incubation at 4 °C (overnight). The following day, the plate was washed, and biotinylated detection antibody solution was added for 30 min at room temperature with shaking. The plate was then incubated with streptavidin–phycoerythrin for 30 min at room temperature with shaking. After additional washing, a reading buffer was added to the plate prior to run in a Luminex Bio‐Plex 200 system (Bio‐Rad). Samples with a bead count <50 were discarded from analysis. Results were analyzed using the ProcartaPlex Analysis App (ThermoFisher).

### ELISpot Immunoassay for Analysis of IL1*β* Secretion by Human Macrophages

A Human IL1*β* ELISpot immunoassay was performed using a Human IL1*β* ELISpotPLUS kit (Mabtech) following manufacturer's instructions. Pre‐coated (mAb‐MT175) plates were washed and blocked for 1 h using RPMI (10% FBS) medium. Human macrophages were washed, counted, and resuspended in RPMI (10% FBS) medium; 4 × 10^3^ macrophages/well were added to the plate in the absence of LPS and incubated for 24 h at 37 °C with 5% CO_2_. Next, plates were washed and incubated with biotinylated detection antibody (mAb‐7P10) for 2 h at room temperature. After incubation, plates were washed and incubated with streptavidin–alkaline phosphatase for 1 h at room temperature. For color development, plates were washed and incubated with the substrate solution containing 5‐bromo‐4‐chloro‐3‐indolyl phosphate/nitro blue tetrazolium for 10 min at room temperature, followed by extensive washing to stop color development. Developed plates were dried overnight before image acquisition using a CTL ImmunoSpot S6 Ultra‐V Analyzer (Software Version 7.0.26.0). Spot size gate limits were established with negative and positive control wells using the “automated gating” tool to determine spot number and size for each condition.

### Zebrafish Lines and Maintenance

Husbandry of adult zebrafish (*Danio rerio*) was performed as previously described.^[^ ^61^ ^]^ WT and transgenic lines including Tg(*kita:HRAS^G12V^‐GFP*) (referred to as Ras),^[^ ^1^, ^28^ ^]^ Tg(*lyz:DsRed*),^[^ ^40^ ^]^ Tg(*mpeg1:mCherry*),^[^ ^39^ ^]^ Tg(*mpeg1:FRET*) (kind gift from Nikolay Ogryzko and Stephen Renshaw, Sheffield, UK), Tg(*lyz:TagRFP‐miR223sponge*), Tg(*mpeg1:eGFP‐miR223sponge*), Tg(*lyz:miR223‐TagRFP*), Tg(*mpeg1:miR223‐eGFP*), Tg(*lyz:TagRFP*), Tg(*mpeg1:eGFP*), and Tg(*il1β:GFP*)^[^ ^43^ ^]^ were maintained on Tüpfel long fin (TL), Ekkwill (EKK) WT or casper^[^ ^62^ ^]^ background. The miR223KO line^[^ ^19^ ^]^ was maintained on WT background or used in combination with transgenic lines. The miR223KO line was genotyped by PCR, using 5′‐CAGGAGGAAGAGGGAGGAGTAA‐3′ (Forward) and 5′‐AATGAATGTTGTCATCCTCCACA‐3′ (Reverse) primers, followed by digestion (*HincII*).

### Generation of Transgenic Zebrafish Lines

The neutrophil‐ and macrophage‐specific miR223sponge lines (Tg(*lyz:TagRFP‐miR223sponge*) and Tg(*mpeg1:eGFP‐miR223sponge*)) were generated using *pTol2‐lyz:TagRFP‐miR223sponge* and *pTol2‐mpeg1:eGFP‐miR223sponge* constructs, respectively, which were made with Tol2kit multisite Gateway cloning technology.^[^ ^63^ ^]^

To generate *pTol2‐lyz:TagRFP‐miR223sponge*, *pTol2‐krt4:TagRFP‐miR223sponge* (Addgene #97150)^[^ ^19^ ^]^ containing a sequence with six copies of bulged miR223 binding sites inserted downstream of TagRFP was first combined with a donor vector (*pDONR221*) to produce middle entry clone vector *pME‐TagRFP‐miR223sponge* in a BP Gateway reaction using BP clonase (Invitrogen). *pME‐TagRFP‐miR223sponge* was then combined with 5′ entry clone vector *p5E‐lyz*, 3′ entry clone vector *p3E‐polyA* and destination vector *pDestTol2pA2* to make *pTol2‐lyz:TagRFP‐miR223sponge* in a LR Gateway reaction using LR clonase (Invitrogen).

To generate *pTol2‐mpeg1:eGFP‐miR223sponge*, *pME‐eGFP‐miR223sponge* was first made from *pME‐TagRFP‐miR223sponge*, replacing TagRFP by eGFP, using a Phusion site‐directed mutagenesis kit (ThermoFisher). *pME‐eGFP‐miR223sponge* was then combined with *p5E‐mpeg1*, *p3E‐polyA*, and *pDestTol2pA2* to make *pTol2‐mpeg1:eGFP‐miR223sponge* in a LR Gateway reaction.

Controls for miR223sponge lines (Tg(*lyz:TagRFP*) and Tg(*mpeg1:eGFP*)) were generated using *pTol2‐lyz:TagRFP* and *pTol2‐mpeg1:eGFP*, respectively, both expressing the empty backbone. These constructs were generated following the protocol described above, using *pME‐TagRFP* (made from *pTol2‐krt4:TagRFP*, Addgene #97149)^[^ ^19^ ^]^ and *pME‐eGFP* (made from *pME‐TagRFP*).

The neutrophil‐specific miR223 overexpression line (Tg(*lyz:miR223‐TagRFP*) was generated using *pTol2‐lyz:miR223‐TagRFP* (Addgene #97148).^[^ ^19^ ^]^

The macrophage‐specific miR223 overexpression line (Tg(*mpeg1:miR223‐eGFP*)) was generated using *pTol2‐mpeg1:miR223‐eGFP*, and this construct was made from *pTol2‐lyz:miR223‐TagRFP*, replacing lysozyme (lyz) and TagRFP by mpeg1 and eGFP, respectively, using a Phusion site‐directed mutagenesis kit.

Controls for miR223 overexpression lines (Tg(*lyz:TagRFP*) and Tg(*mpeg1:eGFP*)) were generated using *pTol2‐lyz:TagRFP* and *pTol2‐mpeg1:eGFP*, respectively, both expressing the empty backbone. These constructs were generated by deleting miR223 from *pTol2‐lyz:miR223‐TagRFP* and *pTol2‐mpeg1:miR223‐eGFP*, with traditional cloning (digestions and ligations).

Sequences of all constructs were confirmed by digestion and sequencing.

### Construct Microinjection in Zebrafish Embryos

For transgenic line generation, 0.5–1 nL of mixture containing 250 ng DNA construct and 500 ng Tol2 mRNA were injected into the cytoplasm of one‐cell stage embryos, in TL or EKK WT background, as previously described.^[^ ^64^ ^]^ Injected larvae were selected for TagRFP/eGFP‐positive neutrophils/macrophages at 5 days post injection by fluorescent microscopy, grown to sexual maturity, and screened for germline transmission. Multiple positive founders for each line were selected to grow successive generations and F3 fish were used for experiments.

### PC Injection in Zebrafish

Larvae were anaesthetized at 2 dpf in MS‐222 (Sigma) and injected systemically with 2 nL of PCs (PC concentrations = 1.25 × 10^7^ [high], 5 × 10^6^ [medium], and 2.5 × 10^6^ [low] PCs/µL) or media alone into the caudal vein using a glass needle, as previously described.^[^ ^65^ ^]^ Local injection was performed subcutaneously with 1 nL of PCs (PC concentration = 1.25 × 10^7^ PCs/µL [high]) or media alone in the somite directly adjacent the cloaca of 3 dpf zebrafish larvae using a glass needle, as previously described.^[^ ^66^ ^]^ When multiple local injections were performed, PCs were injected in the same somite on consecutive days (3, 4, and 5 dpf). Cancerous larvae with similar numbers of pre‐neoplastic cells were selected for injection at 3 dpf. In all larval experiments, anti‐miRs were used at 5 µm (in bulk solution containing 1.25 × 10^7^ PCs/µL), unless otherwise indicated.

1‐year‐old adult fish with similarly sized tailfin tumors (0.2 × 10^7^–3.2 × 10^7^µm^2^) were anaesthetized in MS‐222, and injected intratumorally with single/multiple doses of PCs (20 µL/tumor) using a 30‐gauge insulin syringe (BD Micro‐Fine). In all adult experiments, anti‐miRs were used at 5 µm (in bulk solution containing 5 × 10^5^ PCs/µL).

### RNA Extraction, cDNA Synthesis, RT‐PCR, and qPCR

Total RNA from zebrafish (sorted neutrophils/macrophages) or human (cultured macrophages) cells was extracted with mirVana miRNA isolation kit (Invitrogen). miRCURY LNA RT kit (Qiagen) and Maxima first strand cDNA synthesis kit (ThermoFisher) were used to synthesize cDNA from extracted miRNAs and mRNAs, respectively. RT‐PCR was performed with fast cycling PCR kit (Qiagen) in a PTC‐200 thermal cycler. qPCR was performed with PowerUp SYBR green master mix (ThermoFisher) and run in a QuantStudio 3 real‐time PCR system. Data from qPCR were normalized to the indicated housekeeping genes/miRs. Primer sequences used for miRNA amplification were Human‐miR223‐3p (Qiagen, YP00205986), Zebrafish‐miR223‐3p (Qiagen, YP00205120), Human‐miR142‐3p (Qiagen, YP00204291), Human/Zebrafish‐miR92a‐3p (Qiagen, YP00204258), and Human‐anti‐miR223 (5′‐CGCAGTGGGGTATTTGA‐3′ [Forward], 5′‐TCCAGTTTTTTTTTTTTTTTGTCAGT‐3′ [Reverse], designed with miRprimer software^[^ ^67^ ^]^), and for mRNA amplification are listed in Table S1, Supporting Information.

### Zebrafish Tissue Preparation for FACS

Cell suspensions from adult tailfins were prepared for fluorescence‐activated cell sorting (FACS) using the following method. Dissected adult tailfins were digested with 0.25% trypsin and 5 mg mL^−1^ collagenase type 2 (Worthington Biochemical), 1 h at 32 °C with agitation. Digestion was stopped with 10% FBS, disassociated cells were filtered through a 40 µm Falcon Cell Strainer (ThermoFisher) and resuspended in 2% FBS. Single, live, and positive cells were sorted with a BD influx fluorescence associated cell sorter for downstream RNA protocols. The quality of sorting was demonstrated by RT‐PCR of specific cell markers.

### Live Staining of Zebrafish and Human Macrophages

To visualize PCs internalized by human macrophages, cells were harvested on day 6 of culture and transferred to µ‐Slide 4‐well Ph+ plates (Ibidi) for imaging at a density of 2.6 × 10^5^ cells/well. On day 7, cells were stained with 1 µm CellTracker red CMTPX dye (ThermoFisher) for 2 h before PC supplementation.

To visualize internalized PCs targeted to macrophage lysosomes, zebrafish larvae (3 dpf) with fluorescently labeled macrophages or human macrophages (day 7) previously stained with CellTracker, were treated with PCs for 24 h, and then incubated with 10 µm (1 h) or 75 nm (2 h) LysoTracker deep red (ThermoFisher), respectively, prior to imaging.

### Immunohistochemistry of Cryosections

Adult tumors were dissected, promptly embedded in Tissue‐Tek O.C.T. (ThermoFisher), frozen in isopentane, and 10‐µm transverse sections cut with a Bright OTS cryostat.

For tumor volume or pigmentation measuring experiments, unprocessed cryosections were directly imaged using a Leica MZ10 F Stereomicroscope or a Leica TCS SP8 AOBS confocal laser scanning microscope using 10× air lens, respectively.

For immunohistochemistry, cryosections were washed with 0.5% PBST (Triton X‐100 in PBS, room temperature) prior to blocking with 10% Goat serum (3 h, room temperature) and incubation with primary antibody (overnight, 4 °C). The following day, sections were washed, blocked again, and incubated with secondary antibody (2 h, room temperature). Sections were washed, mounted in Vectashield HardSet antifade mounting medium with 4′,6‐diamidino‐2‐phenylindole (DAPI) (2bscientific) and imaged with a Leica TCS SP8 AOBS confocal laser scanning microscope using 10× air lens. Images of whole sections were processed using Fiji and constructed by “tiling” several maximum projection micrographs together.

Primary antibodies used included Chicken polyclonal anti‐L‐plastin^[^ ^7^ ^]^ (1:500), Mouse monoclonal anti‐IL1*β* ^[^ ^68^ ^]^ (1:500, Abmart), and Rabbit monoclonal anti‐pH3^[^ ^29^ ^]^ (1:200, Cell Signalling) antibodies. Secondary antibodies used included AlexaFluor‐647 Goat anti‐Chicken (1:200, ThermoFisher), AlexaFluor‐488 Goat anti‐Chicken (1:200, ThermoFisher), AlexaFluor‐488 Goat anti‐Mouse (1:200, ThermoFisher), and AlexaFluor‐633 Goat anti‐Rabbit (1:500, ThermoFisher) antibodies.

### TUNEL Staining of Cryosections

Tumor sections were obtained as described above, and TUNEL staining was performed using ApopTag red in situ apoptosis detection kit (Sigma). In brief, cryosections were incubated with 1% PFA (10 min, room temperature), ethanol:acetic acid (2:1, 5 min, −20 °C), equilibration buffer (30 min, room temperature), TdT Enzyme (1 h, 37 °C), and stop/wash buffer (10 min, room temperature). Blocking solution (2 h, room temperature) and anti‐digoxigenin conjugate (with rhodamine, overnight, 4 °C) were added to sections. The following day, sections were washed, mounted, and imaged as described above.

### Live Imaging of Zebrafish and Human Macrophages

Anaesthetized larvae were mounted in 1% low‐melting agarose (Sigma) in glass‐bottomed dishes with Danieau's solution and MS‐222, and imaged using a Leica TCS SP8 AOBS confocal laser scanning microscope with 20× glycerol lens, at 28 °C. In timecourse experiments, fish were kept in dishes with Danieau's solution, and imaged when required. Images were processed using Fiji, and displayed as maximum projections (or single z‐slice images in Figure 2D and Figure S3B, Supporting Information).

Anaesthetized juveniles and adults were placed in dishes with system H_2_O and MS‐222, and imaged using a Leica MZ10 F Stereomicroscope. In timecourse experiments, fish were kept in individual tanks and imaged when required.

Human macrophages were imaged on Ibidi plates using a Leica SP8 AOBS confocal laser scanning microscope with 40× oil lens, at 37 °C.

Movies generated from timelapse imaging experiments were exported from Fiji as QuickTime movies. For 3D reconstructions, imaging data were processed using Imaris (Version 7.6.5).

### Post‐Image Analysis

All image analysis was performed in Fiji.^[^ ^69^ ^]^ Detection, tracking, and spatial analysis of cells/PCs used the modular image analysis (MIA) automated workflow plugin for Fiji.^[^ ^70^ ^]^ The numerical values for the settings were derived empirically and chosen to accurately represent the fluorescence signal. The following post‐image quantifications (Q) were performed:

- *(Q1) Pigmentation*: Pigmentation of zebrafish tailfins/tumors was automatically quantified by applying the threshold function in Fiji to images of these regions, as previously described.^[^ ^29^ ^]^
- *(Q2) Pre‐neoplastic cells and il1β‐expressing macrophages/neutrophils* : Pre‐neoplastic cells were automatically quantified using fluorescent pixel count analysis, as previously described,^[^ ^65^ ^]^ from images of adult tailfins or by manual counting from images of larvae. il1*β*‐expressing macrophages/neutrophils in larvae were also manually counted.
- *(Q3) Tumor area*: Tumor area was quantified by outlining the tumoral mass margins with Fiji after visualization of the bright‐field channel. GFP channel was used to confirm the Ras expression in the outlined tumor region. For timecourse experiments, percentage of tumor area for each fish was calculated relative to the first timepoint.
- *(Q4)* *PC* *speed*: Speed of free‐circulating PCs in an intersegmental artery (above cloaca) was automatically quantified from movies of 3000 frames. PCs were detected and tracked between frames using the TrackMate plugin for Fiji.^[^ ^71^ ^]^ The tracking step was modified to favor linking tracks moving from left to right, with a maximum permitted deviation of ±70° from this orientation. PC tracks were used to calculate instantaneous velocity.
- *(Q5)* *PC* *uptake by immune cells*: PCs inside/outside immune cells were automatically quantified from z‐stack images using the following method. Raw images were optionally passed through a 2D median filter to remove noise and binarized with an Otsu threshold.^[^ ^72^ ^]^ Prior to threshold application, the calculated threshold was systematically adjusted with a user‐controlled multiplication factor and subject to a minimum permitted threshold to prevent segmentation of background. Objects were identified as contiguous foreground‐labeled regions.^[^ ^73^ ^]^ Detected cells smaller than a user‐defined threshold volume were removed from further analysis. Depending on the experiment, immune cells were separated into two groups: those containing PCs and those without.
- *(Q6) Immune cells*: Immune cells were automatically quantified from z‐stack images. Immune cells were detected using a similar method as in (Q5), except with a Gaussian filter to reduce noise and with detected cells falling outside an optional region of interest removed from the final analysis.
- *(Q7)* *PC* *s within macrophage lysosomes*: PCs inside/outside macrophage lysosomes were manually quantified from fish, or different fields of view in each well of cultured macrophages (from at least 400 PC‐containing cells per experiment). Positive events were counted when PCs fully colocalized with lysosomes.
- *(Q8) Intact* *PC* *s*: PCs with/without anti‐miR223 were automatically quantified from z‐stack images using same method as in (Q5).
- *(Q9) Anti‐miR223* *uptake by macrophages*: Anti‐miR223 inside/outside macrophages was automatically quantified from z‐stack images using same method as in (Q5).
- *(Q10) Immunohistochemistry*: L‐plastin‐IL1*β*‐double positive, pH3‐positive, or TUNEL‐positive cells were automatically quantified from immunostained tumor sections. Middle sections from each tumor were selected for immunostaining. Tumor sections were automatically identified following application of variance and median filters to the raw image. The resulting image was binarized using the mean thresholding method^[^ ^74^ ^]^ and binary hole‐filling used to refine the segmented section prior to detection via connected‐components labeling.^[^ ^73^ ^]^ Cells were detected in images which had undergone an optional 2D median filter, followed by rolling‐ball background subtraction and Otsu‐based thresholding. Applied thresholds were subject to a systematic multiplier value and minimum value. Optionally, adjacent cells were split using the watershed method and only those larger than a user‐defined threshold and within the previously detected region of interest, retained for analysis.
- *(Q11) Tumor volume*: Tumor volume was determined by automatic quantification of the tumor area from sections as in (Q10), albeit with the initial variance filter replaced by rolling ball background subtraction. Visualization of the GFP channel was used to confirm Ras expression in all tumor sections. Tumor area of each section was multiplied by the section thickness (10 µm) to obtain the tumor volume of individual sections, and total tumor volume was calculated by the sum of all these volumes, as previously described.^[^ ^75^ ^]^

The software used in this study for post‐image analysis with Fiji, the MIA plugin, and the corresponding workflows for quantification, unless otherwise referenced, were developed by S.J.C. and can be found at https://zenodo.org/record/6304799.

### Statistical Analysis

Statistical analyses and generation of graphics were performed using GraphPad Prism 9 (Version 9.1.0). Details of statistical tests used are indicated in the respective figure legends. Data were confirmed to be normally distributed via D'Agostino–Pearson omnibus or Shapiro–Wilk tests prior to further comparisons. When the data were normally distributed, unpaired two‐sided *t*‐test or ordinary one‐way ANOVA test with Bonferroni's multiple comparison post‐test were used to compare two groups or more than two groups, respectively. For non‐normally distributed data, unpaired two‐sided Mann–Whitney test or Kruskal–Wallis test with Dunn's multiple comparison post‐test were used for comparison between two groups or more than two groups, respectively. Temporal differences between curves in *XY* graphs were compared with two‐way ANOVA test, and Bonferroni's multiple comparison post‐test was performed to compare differences at set timepoints between these curves. Fisher's exact test was used in the analysis of contingency tables to compare proportions between two groups. Statistical significance is indicated on graphs using standard conventions, as follows: non‐significant (ns), *p* ≥ 0.05, *p < 0.05, **p < 0.01, ***p < 0.001, ****p < 0.0001. Sample size used in the experiments and numbers of independent experiments are indicated on graphs, and in the figure legends. Details of data presentation (e.g., meaning of horizontal bars, error bars, data points, significance symbols) for each graph are included in the figure legends.

### Ethics Statement

The zebrafish studies were reviewed and approved by the University of Bristol Animal Welfare and Ethical Review Body (AWERB), and were carried out under UK HO license number PPL PP3332518. For the human primary cell work, PBMNCs were isolated from platelet apheresis blood waste (NHSBT, Bristol, UK) from anonymous healthy donors with informed consent. Ethics approval for all experimental protocols with these cells was granted by Bristol Research Ethics Committee (REC 12/SW/0199), and methods were carried out in accordance with approved guidelines.

## Conflict of Interest

The authors declare no conflict of interest.

## Author Contributions

P.L.‐C. and C.X. contributed equally to this work. S.M. and P.M. jointly supervised this work. P.L.‐C., S.M., and P.M. conceived and designed the study. P.L.‐C. performed zebrafish injection, staining, and imaging experiments. P.L.‐C. performed RT‐PCR and qPCR experiments from zebrafish and human samples. P.L.‐C. performed imaging experiments and Luminex and ELISpot immunoassays of human macrophages. P.L.‐C. generated and characterized the zebrafish transgenic lines Tg(*lyz:TagRFP‐miR223sponge*), Tg(*mpeg1:eGFP‐miR223sponge*), Tg(*lyz:miR223‐TagRFP*), Tg(*mpeg1:miR223‐eGFP*), Tg(*lyz:TagRFP*), and Tg(*mpeg1:eGFP*). C.X. performed protocell synthesis, cargo loading, and imaging experiments. C.E.S., P.L.‐C., T.C.L.O., and A.M.T. designed the in vitro human macrophage experiments. C.E.S. and T.C.L.O. performed the experiments on human macrophage cultures. P.L.‐C., C.X., C.E.S., T.C.L.O., and P.M. analyzed the data. P.L.‐C. and S.J.C. carried out the image analysis. P.L.‐C. and P.M. wrote the manuscript. P.L.‐C., C.X., C.E.S., T.C.L.O., S.J.C., A.M.T., S.M., and P.M. reviewed and edited the manuscript. S.M., P.M., and A.M.T. secured funding and provided project administration, supervision, and resources. All authors contributed to the article and approved the submitted version.

## Supporting information

Supporting Information

Supplemental Movie 1

Supplemental Movie 2

Supplemental Movie 3

Supplemental Movie 4

Supplemental Movie 5

Supplemental Movie 6

Supplemental Movie 7

Supplemental Movie 8

Supplemental Movie 9

Supplemental Movie 10

Supplemental Movie 11

## Data Availability

The data that support the findings of this study are available from the corresponding author upon reasonable request.
