# Supplementary material for: Macrophage Reprogramming with Anti‐miR223‐Loaded Artificial Protocells Enhances In Vivo Cancer Therapeutic Potential
Source: Adv Sci (Weinh). 2022 Oct 31;9(35):2202717. doi: 10.1002/advs.202202717 (PMC9762313; doi:10.1002/advs.202202717)
Supplement: Supplementary file 1 — Supporting Information [file ADVS-9-2202717-s001.pdf]

ADVANCED SCIENCE
Open Access

Supporting Information

for *Adv. Sci.*, DOI 10.1002/advs.202202717

Macrophage Reprogramming with Anti-miR223-Loaded Artificial Protocells Enhances In Vivo Cancer Therapeutic Potential

Paco López-Cuevas, Can Xu, Charlotte E. Severn, Tiah C. L. Oates, Stephen J. Cross, Ashley M. Toye, Stephen Mann and Paul Martin**

Supporting Information

Macrophage Reprogramming with Anti-miR223-Loaded Artificial Protocells Enhances In Vivo Cancer Therapeutic Potential

Paco López-Cuevas[1,7], Can Xu[2,7], Charlotte E Severn[1,3], Tiah CL Oates[1,3], Stephen J Cross[4], Ashley M Toye[1,3], Stephen Mann[2,5,6,8,] and Paul Martin[1,8,*]*

[1]School of Biochemistry, Biomedical Sciences Building, University Walk, University of Bristol, Bristol, BS8 1TD, UK.
[2]Centre for Protolife Research, School of Chemistry, University of Bristol, Bristol, BS8 1TS, UK.
[3]National Institute for Health Research Blood and Transplant Research Unit (NIHR BTRU) in Red Blood Cell Products, University of Bristol, Bristol, BS34 7QH, UK.
[4]Wolfson Bioimaging Facility, Biomedical Sciences Building, University Walk, University of Bristol, Bristol, BS8 1TD, UK.
[5]Max Planck Bristol Centre for Minimal Biology, School of Chemistry, University of Bristol, Bristol, BS8 1TS, UK.
[6]School of Materials Science and Engineering, Shanghai Jiao Tong University, Shanghai, 200240, P. R. China.
[7]These authors contributed equally: Paco López-Cuevas, Can Xu.
[8]These authors jointly supervised this work: Stephen Mann, Paul Martin.
***Correspondence:** s.mann@bristol.ac.uk; paul.martin@bristol.ac.uk

Supporting Figures

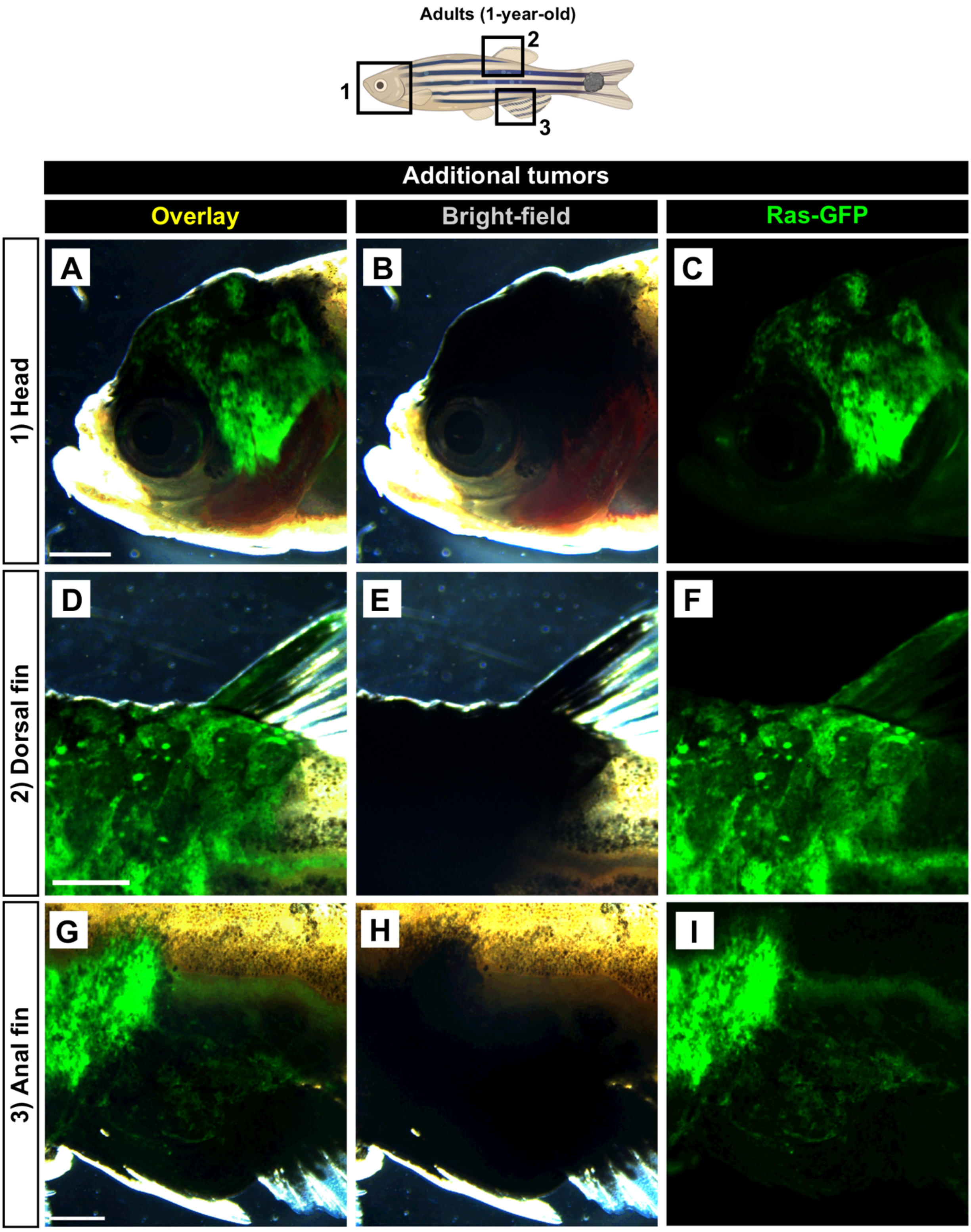

Figure S1. Regions of additional tumor formation in adult zebrafish. A-I) Multi-channel (A,D,G) or single-channel (B,C,E,F,H,I) images showing "secondary" Ras-GFP-positive tumors (green) imaged at different body locations (head [1], dorsal fin [2] or anal fin [3]) in 1-year-old tailfin tumor-bearing fish. Accompanying schematic illustrates fish age and imaged areas (black outlined box) used for the experiment. Scale bars = 1 mm.

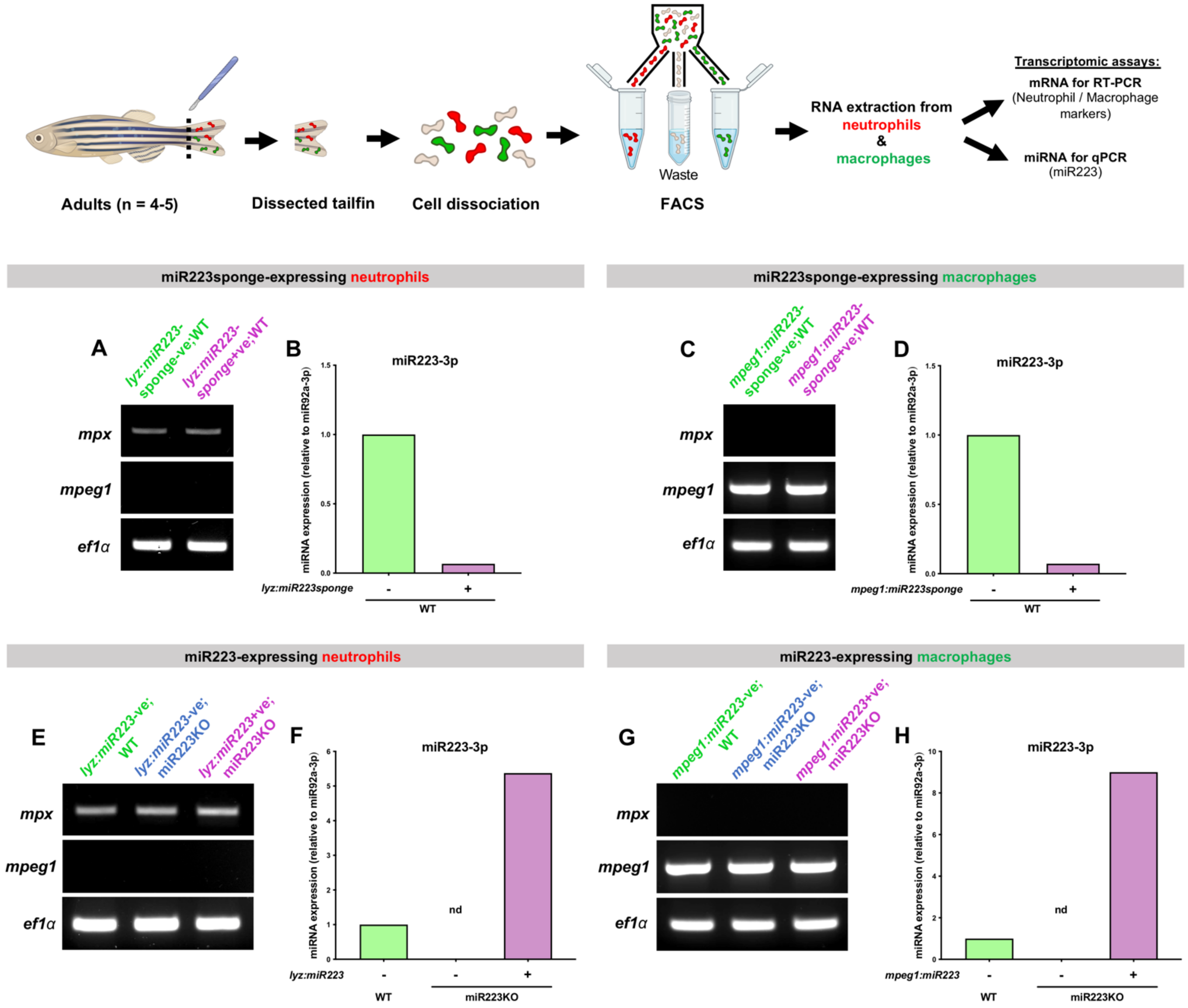

Figure S2. Characterization of neutrophil- and macrophage-specific miR223sponge and miR223 overexpression zebrafish transgenic lines. A-H) Transcriptomic assays from sorted cells used to characterize fish lines expressing miR223sponge or miR223 specifically in neutrophils (A,B,E,F) or macrophages (C,D,G,H). **A,C,E,G)** RT-PCR of mRNA extracted from neutrophils (A,E) or macrophages (C,G) sorted from 4-5 dissected adult tailfins to determine the expression of neutrophil (*mpx*) or macrophage (*mpeg1*) markers in Tg(*lyz:TagRFP;mpeg1:eGFP*);WT (green) and Tg(*lyz:TagRFP-miR223sponge;mpeg1:eGFP-miR223sponge*);WT (magenta) (A,C), or Tg(*lyz:TagRFP;mpeg1:eGFP*);WT (green), Tg(*lyz:TagRFP;mpeg1:eGFP*);miR223KO (blue) and Tg(*lyz:miR223-TagRFP;mpeg1:miR223-eGFP*);miR223KO (magenta) (E,G) fish. *ef1α* was used as loading control. **B,D,F,H)** Bar charts showing qPCR data for the expression levels of miR223-3p in sorted neutrophils (B,F) or macrophages (D,H), from (A,E) or (C,G), respectively. qPCR data were normalized to miR92a-3p (housekeeping miR) from sorted cells from Tg(*lyz:TagRFP;mpeg1:eGFP*);WT (green) fish. Accompanying schematic illustrates the experimental design for sorting cells from dissected adult tailfins and downstream RNA assays. Data are from one experiment. FACS = fluorescence-activated cell sorting; *n* = number of adult fish; nd = not detected.

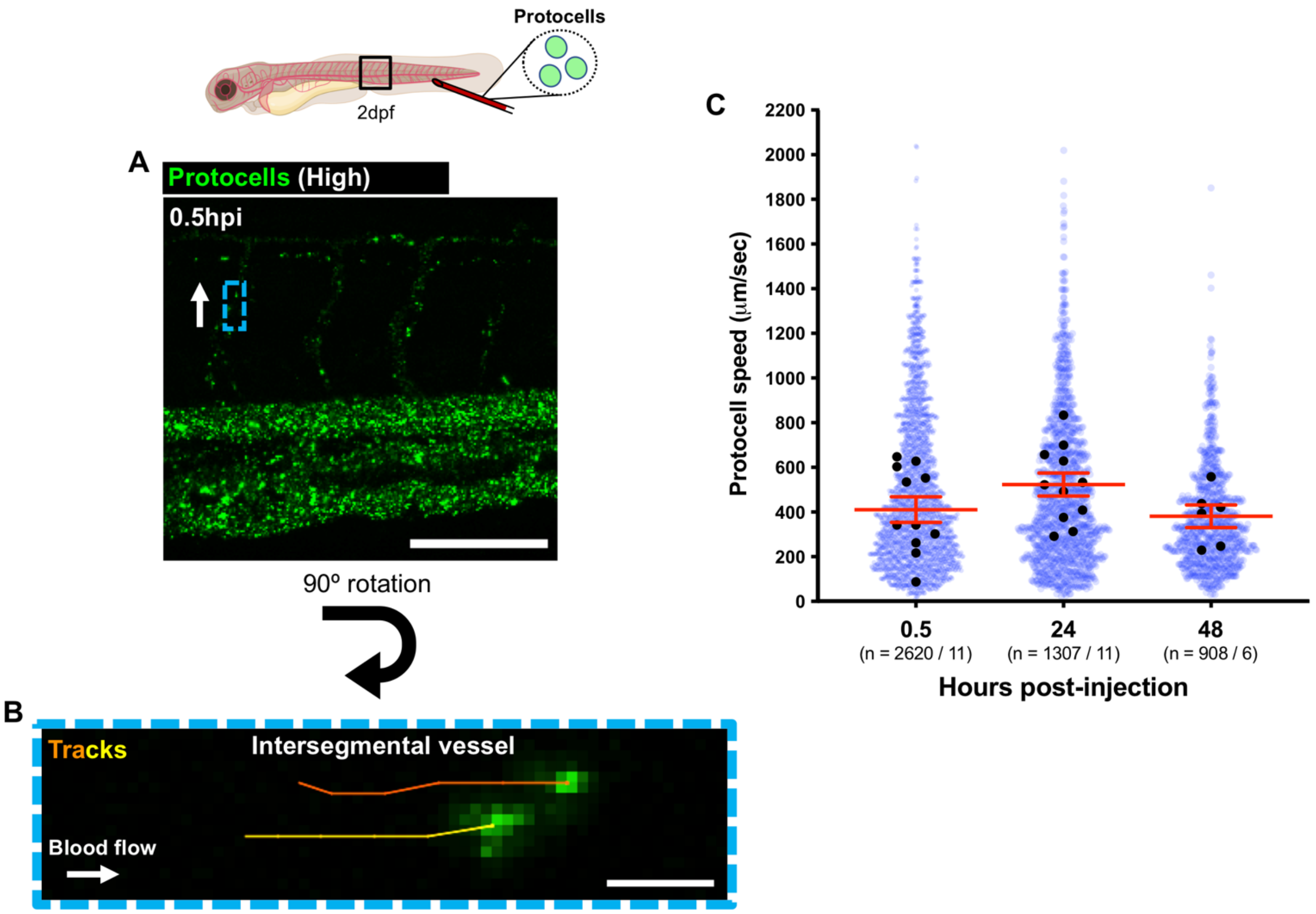

Figure S3. Analyzing the speed of free-circulating protocells. A) Single-channel confocal image (extracted from Figure 2C) of the flank of a 2 dpf casper larva after systemic injection of FITC-protocells at 0.5 hpi; white arrow indicates the direction of the blood flow. **B)** High magnification view of (A) showing two FITC-protocells and their tracks as they move through the intersegmental vessel at 0.5 hpi. See also Movie S2, Supporting Information. **C)** Dot plot showing quantification of protocell speed at 0.5, 24, and 48 hpi from the regions imaged in (B). In these experiments, protocells were injected at a high concentration (1.25×10^7 protocells/µL). Accompanying schematic illustrates developmental stage (larva), type of injection (systemic), and imaged area (black outlined box) used for the experiment. Data are pooled from two independent experiments. Graph (C) shows mean ± SEM, each small dot represents one protocell and larger dots represent the mean from one fish. *n* = number of protocells/fish. Scale bars = 100 µm (A), 5 µm (B).

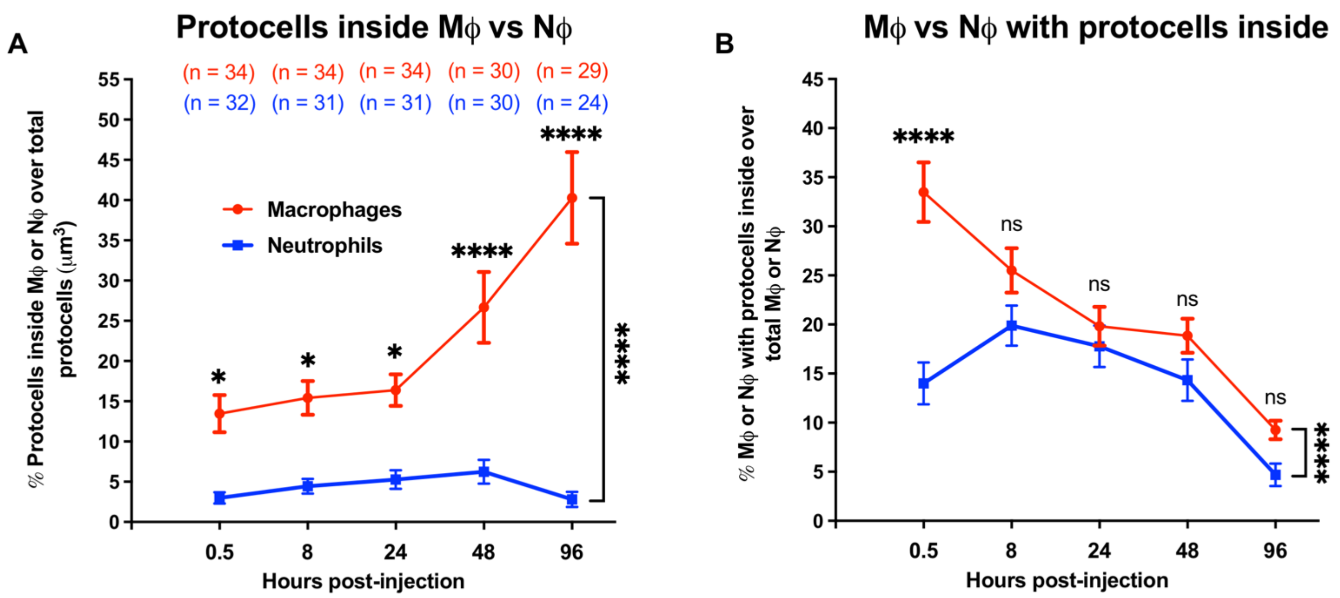

Figure S4. Macrophages are more efficient at taking up free-circulating protocells than neutrophils. A,B) Graphs showing percentage of protocells within leukocytes (A), or percentage of leukocytes containing protocell(s) (B), at the indicated timepoints post injection of systemic FITC-protocells (high concentration = 1.25×10^7 protocells/μL) in 2 dpf Tg(*mpeg1:mCherry*) or Tg(*lyz:DsRed*) larvae. See also Movies S5 and S6, Supporting Information. Data are extracted from Figure 2J-M to enable direct comparison of protocell uptake between macrophages and neutrophils. Data are pooled from three independent experiments and analyzed using two-way ANOVA test with Bonferroni's multiple comparisons test, ns $p \geq 0.05$, *$p < 0.05$, ****$p < 0.0001$. Graphs show mean ± SEM, and each dot represents the mean of all fish analyzed. MΦ = macrophages; *n* = number of fish; NΦ = neutrophils.

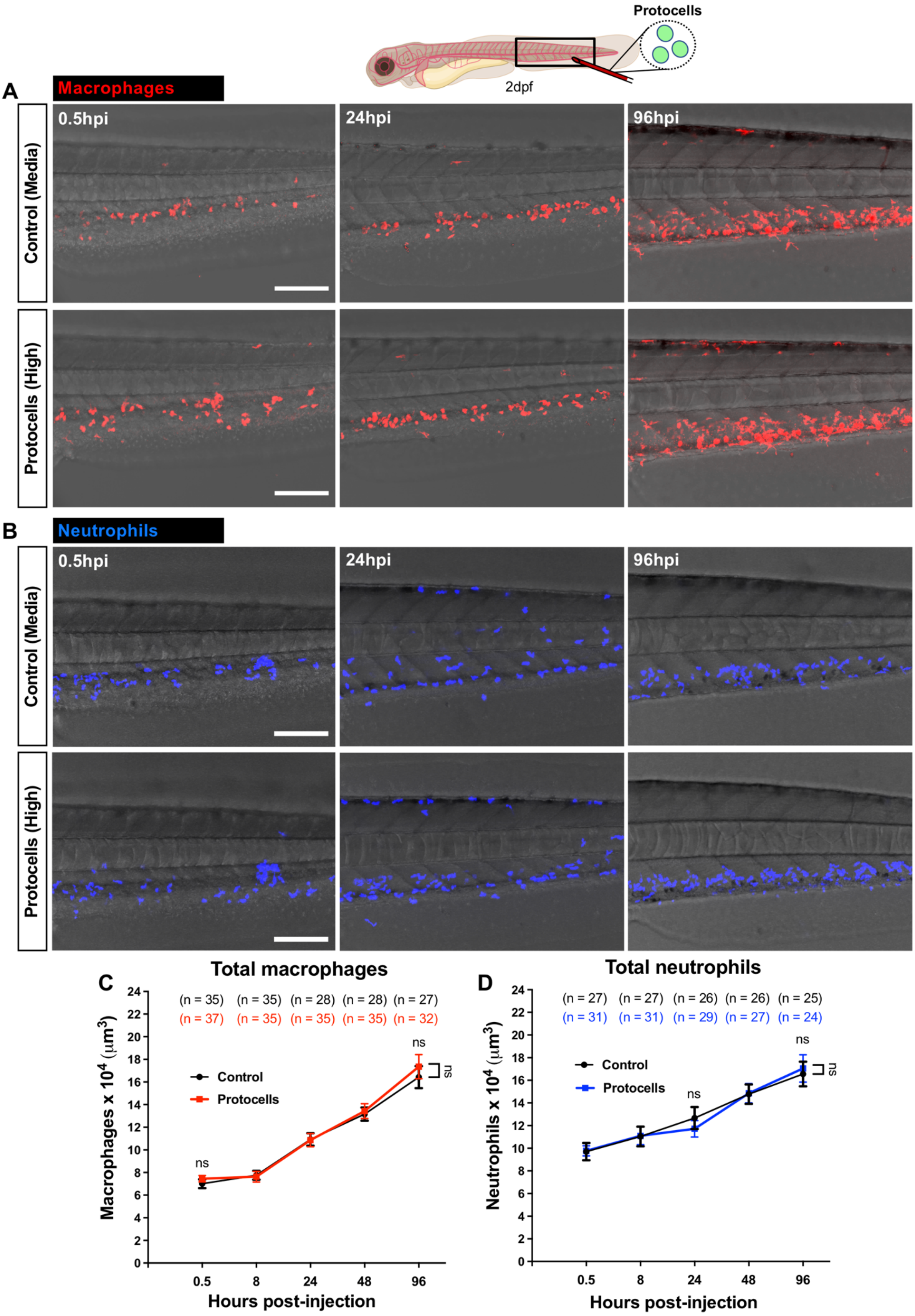

Figure S5. Systemic injection of protocells does not alter leukocyte numbers. A,B) Multi-channel confocal images of the flank of Tg(*mpeg1:mCherry*) (A) or Tg(*lyz:DsRed*) (B) larvae after systemic injection of control (media) or FITC-

protocells at 2 dpf and imaged at 0.5, 24, and 96 hpi to reveal total macrophage and neutrophil numbers. **C,D)** Graphs showing quantification of total macrophages (C) or neutrophils (D) from the regions imaged in (A) and (B) at the indicated timepoints post injection of high protocell concentration (1.25x10^7 protocells/μL). Accompanying schematic illustrates developmental stage (larva), type of injection (systemic), and imaged area (black outlined box) used for the experiment. Data are pooled from three independent experiments and analyzed using two-way ANOVA test with Bonferroni's multiple comparisons test, ns $p \geq 0.05$. Graphs show mean ± SEM, and each dot represents the mean of all fish analyzed. *n* = number of fish. Scale bars = 150 μm.

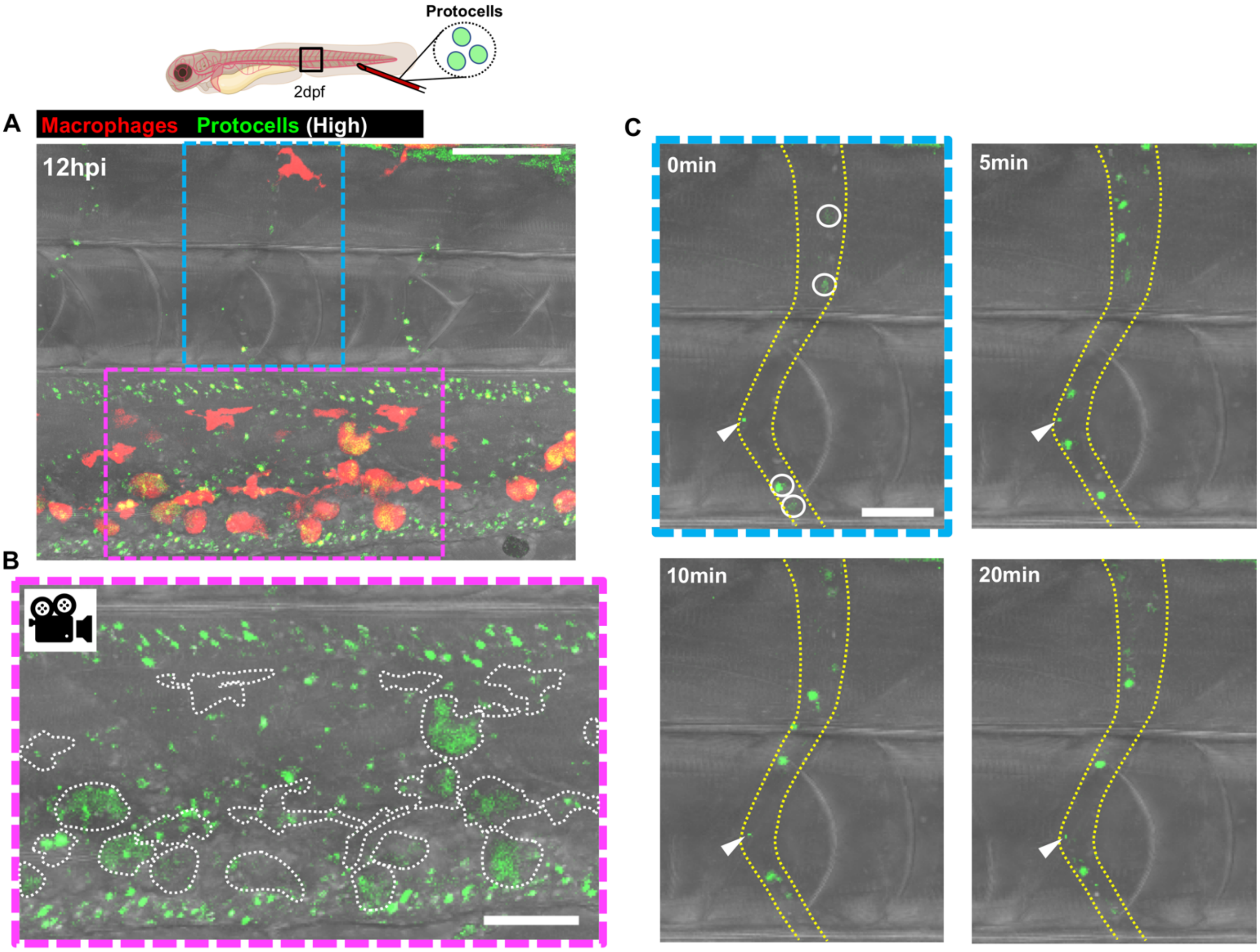

Figure S6. Minimal "trapping" of protocells in the CHT and peripheral vasculature. A) Multi-channel confocal image of the flank of a Tg(*mpeg1:mCherry*) larva after systemic injection of FITC-protocells at 2 dpf and imaged at 12 hpi, to show the distribution of protocells within macrophages and freely circulating in the CHT region (magenta dashed box) and peripheral vessels (blue dashed box). **B)** High magnification view of (A) showing that the majority of FITC-protocells are retained or "trapped" within macrophages (white dotted outlines) at the CHT region while some are still flowing within the vasculature. See also Movie S7, Supporting Information. **C)** High magnification view of (A) showing confocal movie frames of FITC-protocells circulating in an intersegmental vessel (white circles; vessel wall is indicated by yellow dotted lines) or "trapped" in the endothelium (white arrowheads) overtime. See also Movie S8, Supporting Information. "High" corresponds to the protocell concentration injected (1.25×10^7 protocells/μL). Accompanying schematic illustrates developmental stage (larva), type of injection (systemic), and imaged area (black outlined box) used for the experiment. Scale bars = 50 μm (A), 25 μm (B), 20 μm (C).

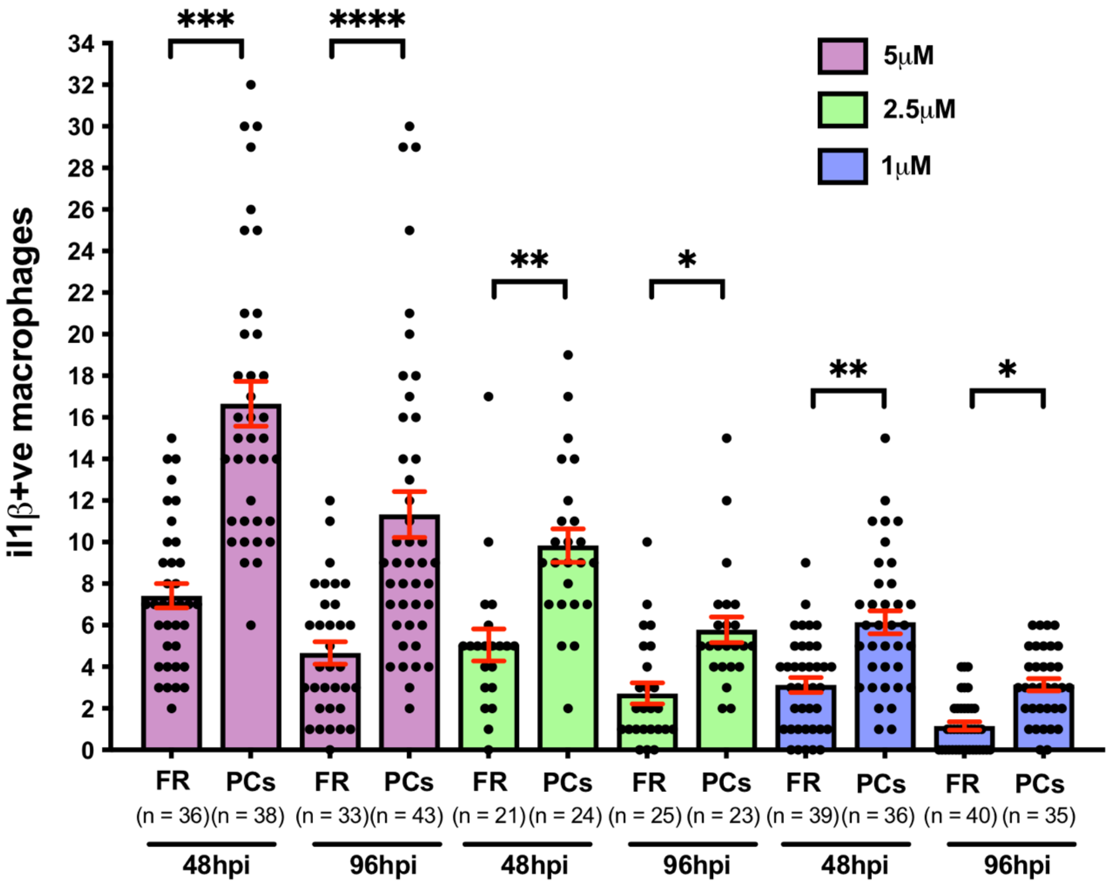

Figure S7. Locally injected anti-miR223 protocells trigger a higher il1β expression in macrophages than free anti-miR223 in a dose dependent manner. Graph showing number of il1β-positive macrophages at two timepoints (48 and 96 hpi) following local injection of 3 dpf Tg(*mpeg1:mCherry;il1β:GFP*) with three different concentrations of unlabeled anti-miR223, free or loaded into protocells. Data are pooled from three independent experiments and analyzed using Kruskal-Wallis test with Dunn's multiple comparisons test, *p < 0.05, **p < 0.01, ***p < 0.001, ****p < 0.0001. Graph shows mean ± SEM, and each dot represents one fish. FR = free anti-miR223; *n* = number of fish; PCs = anti-miR223 protocells.

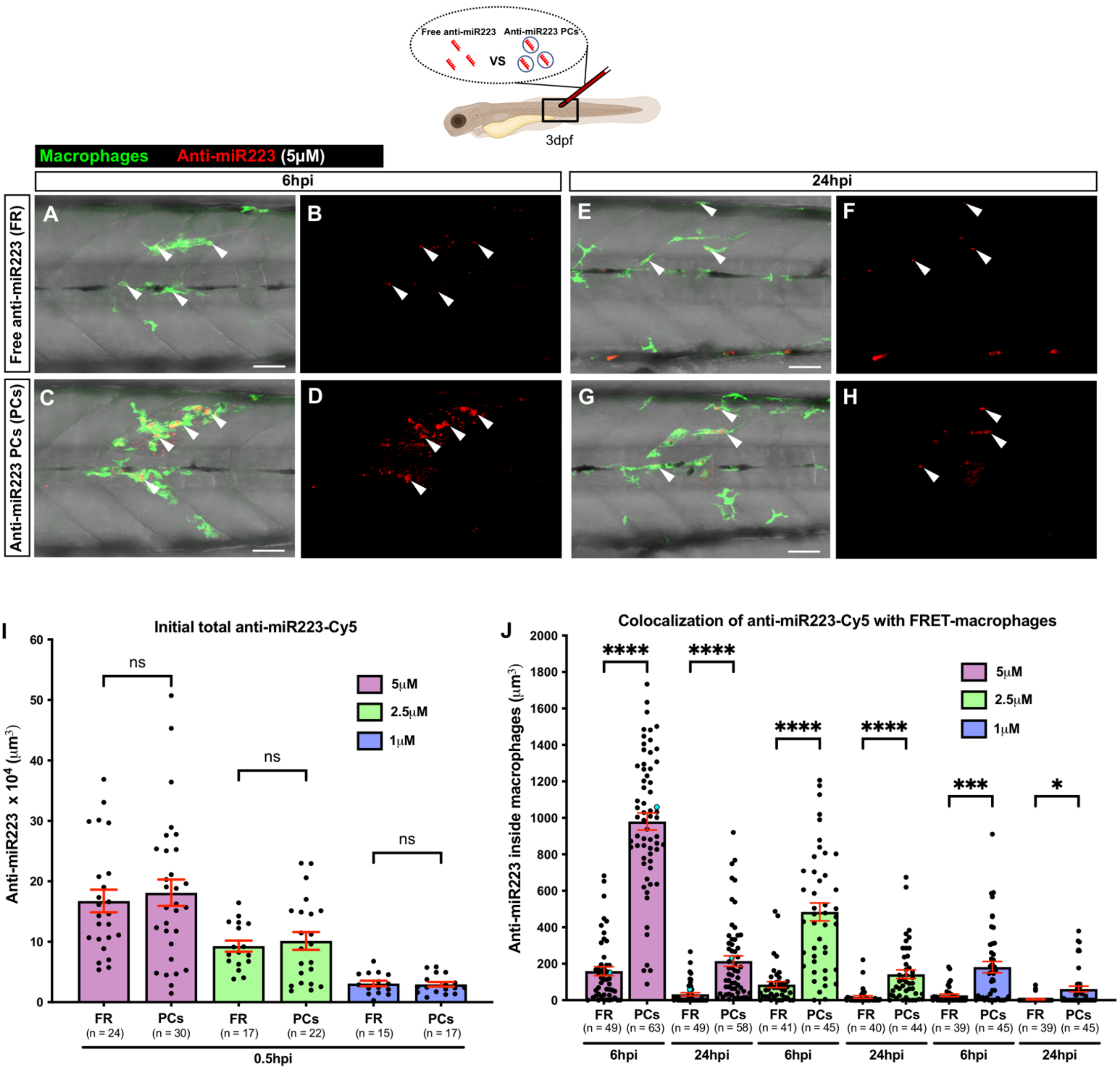

Figure S8. Enhanced anti-miR223 uptake by macrophages via protocell delivery. A-H) Multi-channel (A,C,E,G) or single-channel (B,D,F,H) confocal images of the flank of Tg(*mpeg1:FRET*) larvae after local injection of free anti-miR223-Cy5 or protocells loaded with anti-miR223-Cy5 at 3 dpf and imaged at 6 hpi (A-D) and 24 hpi (E-H); white arrowheads indicate anti-miR223-Cy5 within macrophages. **I)** Graph showing the quantification of total anti-miR223 inside and outside macrophages, immediately after injection (0.5 hpi) of each treatment at the indicated anti-miR223 concentrations from the regions imaged in (A)-(H). Note that no significant difference in total anti-miR223 was observed between free anti-miR223 and anti-miR223 protocell groups at 0.5 hpi, indicating that similar anti-miR223 amounts were consistently injected for each treatment. **J)** Graph showing the quantification of anti-miR223 inside macrophages as quantified by colocalization of Cy5 (anti-miR223) with FRET (macrophages) for each treatment at the indicated timepoints and anti-miR223 concentrations from the regions imaged in (A)-(H). Accompanying schematic illustrates developmental stage (larva), type of injection (local), and imaged area (black outlined box) used for the experiments. Data are pooled from three independent experiments and analyzed using Kruskal-Wallis test with Dunn's multiple comparisons test, ns $p \geq 0.05$, *$p < 0.05$, ***$p < 0.001$, ****$p < 0.0001$. Graphs show mean ± SEM, each dot represents one fish and blue dots correspond to the representative images shown in the panels. FR = free anti-miR223; *n* = number of fish; PCs = anti-miR223 protocells. Scale bars = 50 µm.

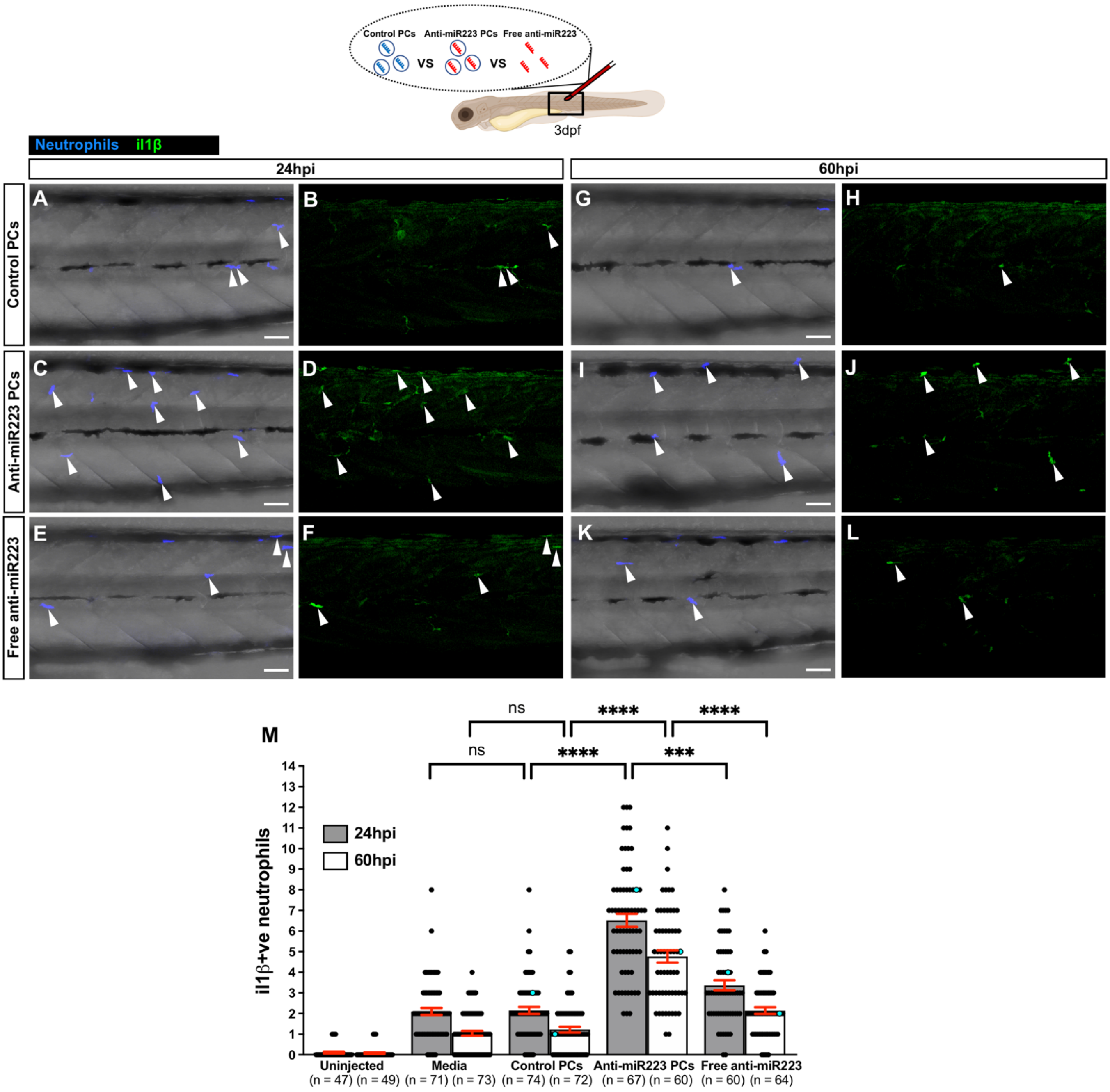

Figure S9. Uptake of anti-miR223 protocells enhances il1β expression in neutrophils. A-L) Multi-channel (A,C,E,G,I,K) or single-channel (B,D,F,H,J,L) confocal images of the flank of Tg(*lyz:DsRed*;*il1β:GFP*) larvae showing il1β-positive neutrophils (white arrowheads) after local injection of unlabeled control protocells, unlabeled anti-miR223 protocells or unlabeled free anti-miR223 at 3 dpf and imaged at 24 hpi (A-F) and 60 hpi (G-L). **M)** Graph showing the number of il1β-positive neutrophils following each treatment quantified from the regions imaged in (A)-(L). Accompanying schematic illustrates developmental stage (larva), type of injection (local), and imaged area (black outlined box) used for the experiment. Data are pooled from three independent experiments and analyzed using Kruskal-Wallis test with Dunn's multiple comparisons test, ns $p \geq 0.05$, ***$p < 0.001$, ****$p < 0.0001$. Graph shows mean ± SEM, each dot represents one fish and blue dots correspond to the representative images shown in the panels. *n* = number of fish; PCs = protocells. Scale bars = 50 μm.

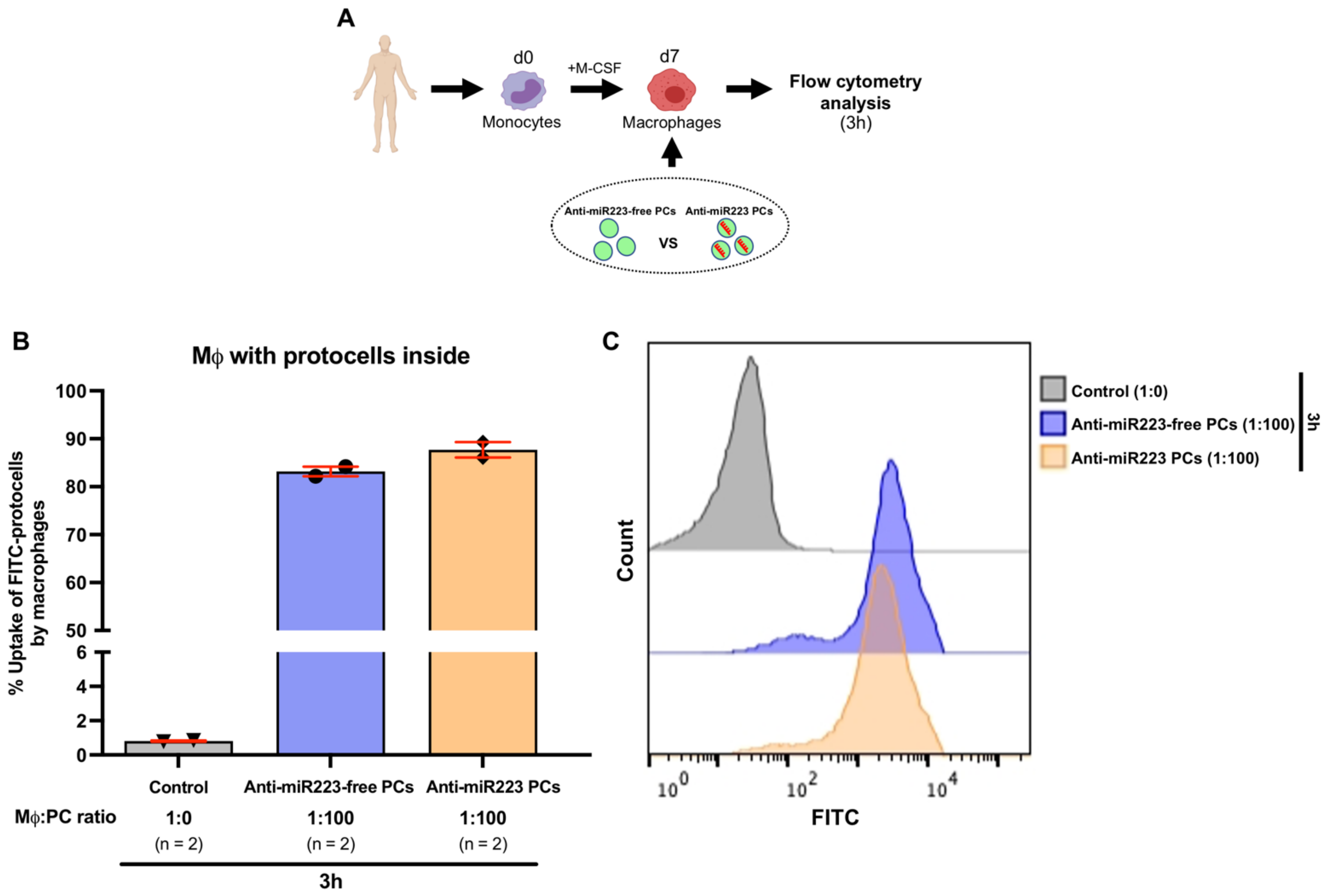

Figure S10. Analyzing the uptake of anti-miR223 protocells by human macrophages in vitro. A) Schematic of the experimental design for evaluation of human macrophage uptake of anti-miR223-loaded protocells ("Anti-miR223 PCs") in comparison with protocells without anti-miR223 cargo ("Anti-miR223-free PCs") through flow cytometry analysis; human macrophages at day 7 of culture were incubated with FITC-protocells, loaded with or without anti-miR223, for 3 h prior to analysis. **B)** Graph showing percentage of human macrophages containing FITC-protocells quantified by flow cytometry after incubation with anti-miR223-free protocells or anti-miR223 protocells for 3 h. **C)** Representative histograms from (B) after each protocell treatment. Data (C) for anti-miR223 protocell group (orange) derives from the same experiment in Figure 6D, and therefore the same data for control (grey) and anti-miR223-free protocell (blue) groups are shown here to enable direct comparison of protocell uptake between macrophages treated with anti-miR223-free protocells and macrophages treated with anti-miR223 protocells. Data are pooled (B) or representative (C) from two independent experiments. Graph (B) shows mean ± SEM, and each dot represents one experiment. MΦ = macrophages; *n* = number of experiments; PC = protocells.

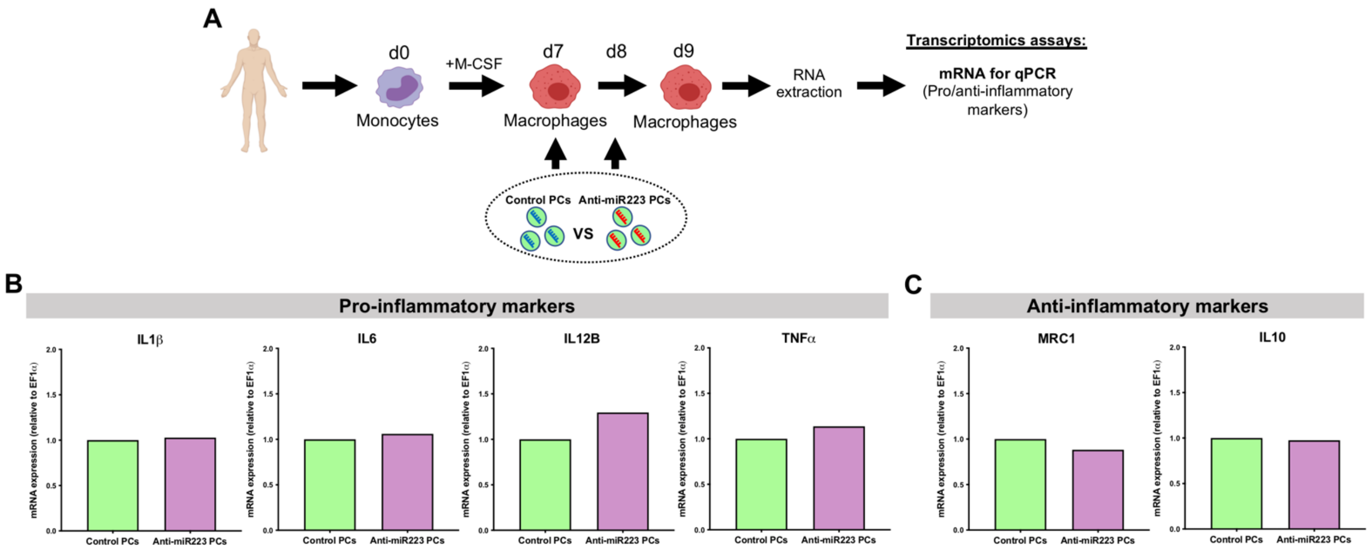

Figure S11. In vitro uptake of anti-miR223 protocells by human macrophages does not affect expression of pro/anti-inflammatory markers in the absence of LPS stimulation. A) Schematic of the experimental timeline to evaluate human macrophage reprogramming (through transcriptomic analysis) after anti-miR223 protocell treatment and in the absence of LPS; FITC-protocells, loaded with control anti-miR or anti-miR223, were administered to macrophage cultures in two consecutive doses (days 7 and 8) prior to total RNA extraction from macrophages (day 9) for qPCR assays. **B,C)** Graphs showing qPCR data for the expression levels of pro-inflammatory markers (IL1β, IL6, IL12B and TNFα) (B) and anti-inflammatory markers (MRC1 and IL10) (C), in human macrophages after each protocell treatment. qPCR data were normalized to EF1α from macrophages treated with control protocells. Data are from one experiment. PCs = protocells.

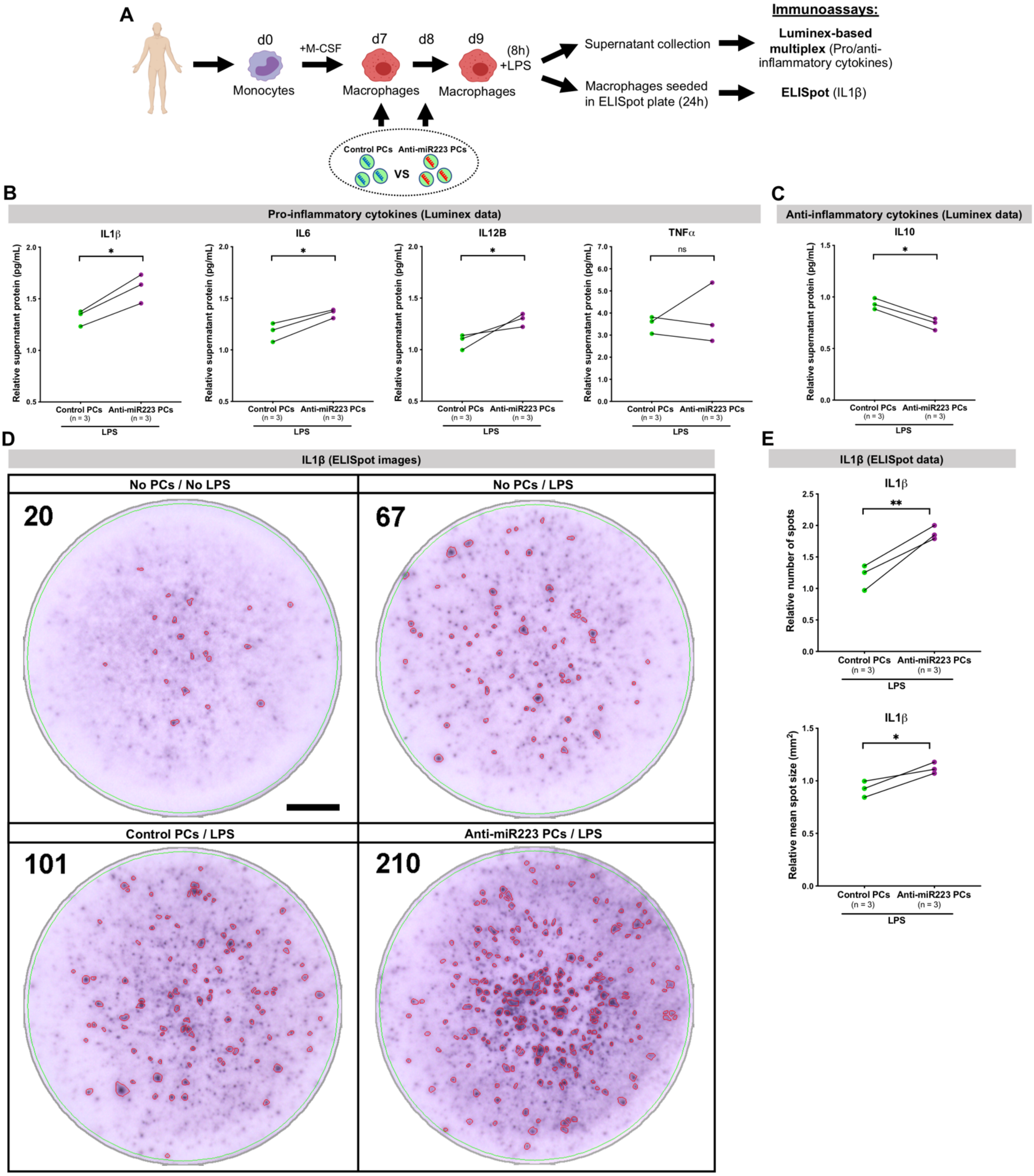

Figure S12. In vitro uptake of anti-miR223 protocells by human macrophages enhances secretion of pro-inflammatory cytokines. A) Schematic of the experimental timeline to evaluate protein secretion (through immunoassays) of reprogrammed human macrophages after anti-miR223 protocell treatment; FITC-protocells, loaded with control anti-miR or anti-miR223, were administered to macrophage cultures in two consecutive doses (days 7 and 8) prior to LPS exposure for 8 h (day 9). Supernatants were immediately collected for protein quantification via Luminex-based multiplex immunoassays, and the LPS-stimulated macrophages were cultured in a human IL1β ELISpot plate for an additional 24-h period (day 10). **B,C)** 5-plex Luminex data showing quantification

of pro-inflammatory cytokines (IL1β, IL6, IL12B and TNFα) (B) and anti-inflammatory cytokines (IL10) (C) present in supernatant of LPS-stimulated human macrophages after each protocell treatment. **D)** Representative images of the wells of an ELISpot plate showing the IL1β spots generated by human macrophages following each protocell treatment and LPS stimulation ("Control PCs/LPS" and "Anti-miR223 PCs/LPS"); unstimulated and LPS-stimulated macrophages without protocell addition ("No PCs/No LPS" and "No PCs/LPS") were used as negative and positive controls, respectively; number of detected spots (red outlines) within the counted area (large green outlines) are shown in the top left corner of each well image. **E)** ELISpot data showing quantification of IL1β secretion, represented by number of spots (top) and mean spot size (bottom) analyzed by ELISpot reader from the images in (D), in human macrophages after each protocell treatment. Luminex and ELISpot data were normalized to LPS-stimulated macrophages that had not been treated with protocells. Data are pooled from three independent experiments and analyzed using unpaired two-sided *t*-test, ns $p \geq 0.05$, $^{*}p < 0.05$, $^{**}p < 0.01$. In all graphs each dot represents one experiment and dots from the same experiment are connected by lines. *n* = number of experiments; PCs = protocells. Scale bar = 1 mm.

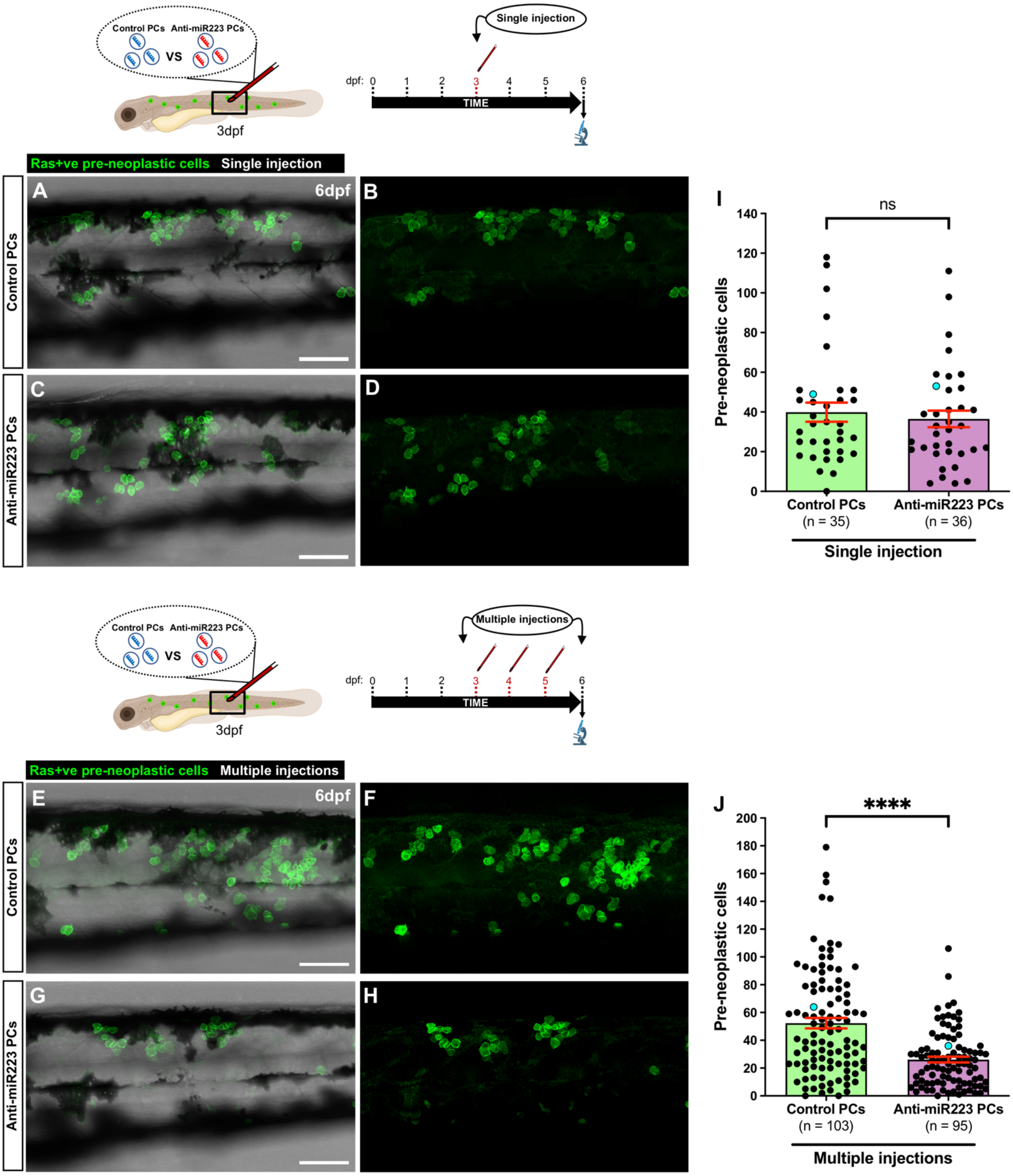

Figure S13. Anti-miR223 protocell treatment leads to reduced pre-neoplastic cell numbers in zebrafish larvae. A-H) Multi-channel (A,C,E,G) or single-channel (B,D,F,H) confocal images of the flank of 6 dpf cancerous Tg(*kita:HRAS*^{G12V}*-GFP*) larvae showing Ras-positive pre-neoplastic cells (green) after a single local injection at 3 dpf (A-D) or multiple injections at 3, 4, and 5 dpf (E-H) of unlabeled control protocells (A,B,E,F) or unlabeled anti-miR223 protocells (C,D,G,H). **I,J)** Graphs showing number of pre-neoplastic cells after each injection regime quantified from the regions imaged in (A)-(H). Accompanying schematics illustrate developmental stage (larva), timeline, type of

injection (local, single or multiple), and imaged area (black outlined box) used for each experiment. Data are pooled from three independent experiments and analyzed using unpaired two-sided Mann-Whitney test, ns $p \geq 0.05$, ****$p < 0.0001$. Graphs show mean ± SEM, each dot represents one fish and blue dots correspond to the representative images shown in the panels. *n* = number of fish; PCs = protocells. Scale bars = 100 μm.

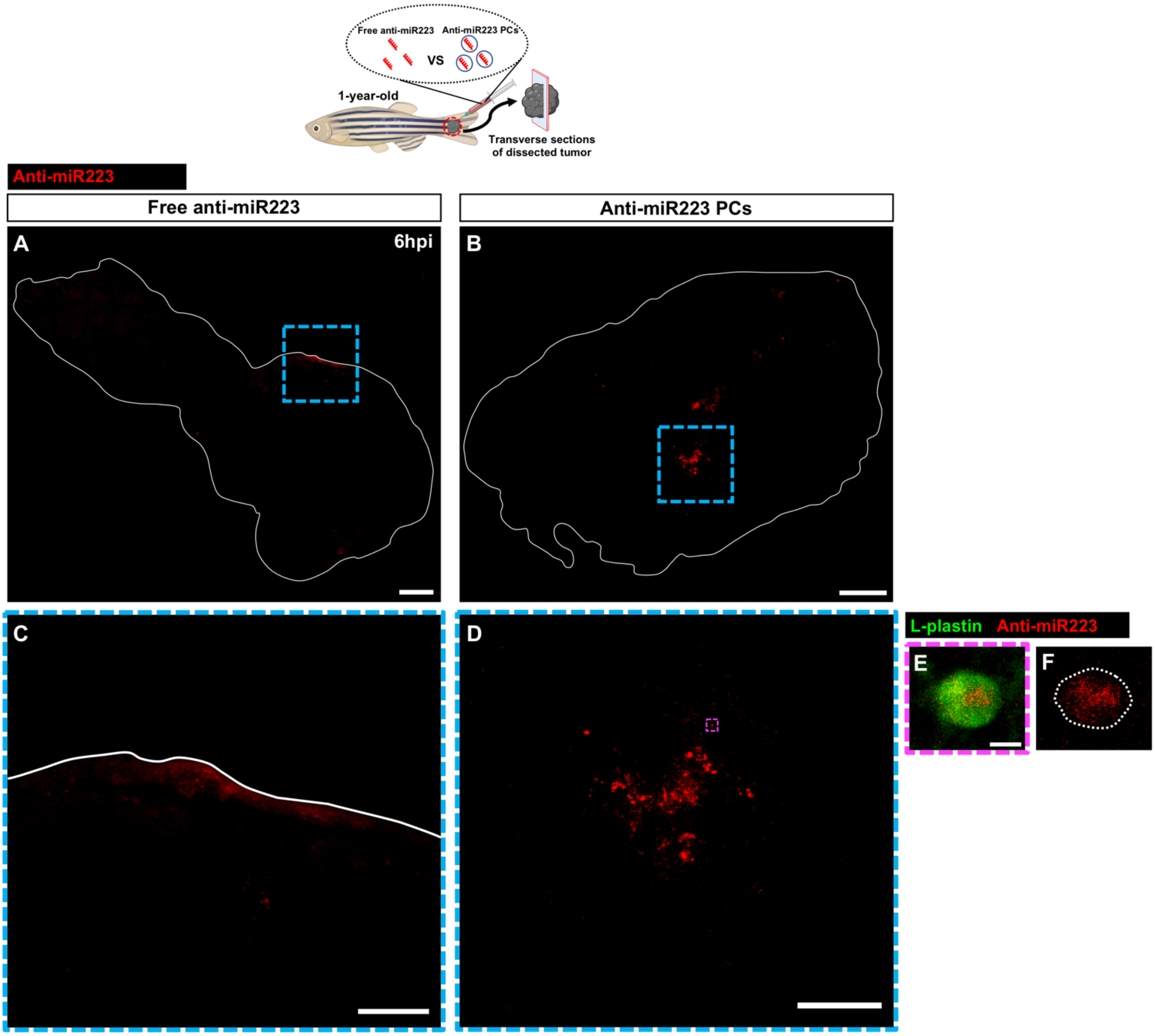

Figure S14. Protocells retain anti-miR223 cargoes and are taken up by leukocytes in the tumor microenvironment whereas free anti-miRs diffuse away from the injection site. A-D) Single-channel confocal images of cryosections from 1-year-old adult tail tumors harvested 6 h after local injection of free anti-miR223-Cy5 (A,C) or unlabeled protocells loaded with anti-miR223-Cy5 (B,D); white lines indicate tumor margins. **C,D)** High magnification views of (A) and (B) showing low levels of anti-miR223-Cy5 at the tumor periphery after diffusing away from injection site (C) or retained at the injected tumor core (D). **E,F)** High magnification views of (D) showing multi-channel (E) or single-channel (F) confocal images of anti-miR223-Cy5 within a L-plastin-positive cell; leukocytes are revealed by anti-L-plastin immunostaining (green) and white dotted outlines indicate leukocyte margins. Accompanying schematic illustrates fish age and type of injection (local) used for the experiment. PCs = protocells. Scale bars = 400 μm (A,B), 150 μm (C,D), 5 μm (E).

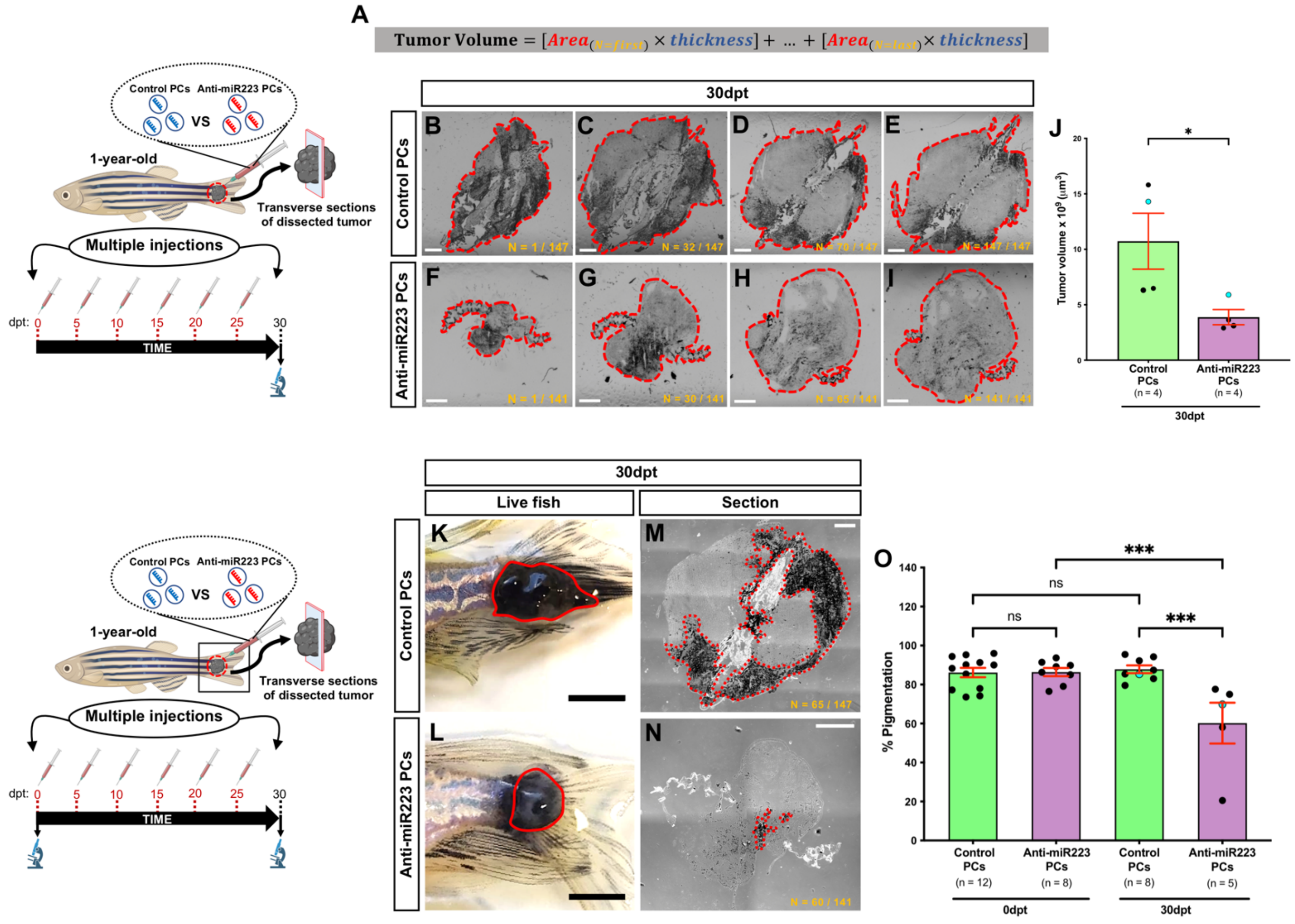

Figure S15. Anti-miR223 protocell treatment leads to a reduction in adult tailfin tumor volume and pigmentation. A) Formula applied to calculate tumor volume from complete transverse histological serial cryosections.[1] **B-I)** Bright-field images of several transverse cryosections from 1-year-old adult tail tumors harvested at 30 dpt after multiple local injections of unlabeled control protocells (B-E) or unlabeled anti-miR223 protocells (F-I); red dashed lines outline the area of the tumor sections used in (A). **J)** Graph showing tumor volume after each protocell treatment calculated using the formula in (A) and regions imaged from (B)-(I). **K,L)** Images showing pigmentation levels of 1-year-old adult tail tumors (red outlines) at 30 dpt after each protocell treatment. **M,N)** Bright-field confocal images showing pigmentation of cryosections from 1-year-old adult tail tumors harvested at 30 dpt after each protocell treatment; areas of high pigmentation are outlined by red dotted lines. **O)** Graph showing percentage of pigmentation at 0 and 30 dpt quantified from the tumor regions imaged in (K) and (L). Accompanying schematics illustrate fish age, timeline, type of injection (local, multiple), and imaged area (black outlined box) used for each experiment. Data are pooled from two independent experiments and analyzed using unpaired two-sided *t*-test (J) or one-way ANOVA test with Bonferroni's multiple comparisons test (O), ns $p \geq 0.05$, *$p < 0.05$, ***$p < 0.001$. Graphs show mean ± SEM, each dot represents one fish and blue dots correspond to the representative images shown in the panels. *n* = number of fish; *N* = section number/total number of sections. Scale bars = 500 μm (B-I,M,N), 5 mm (K,L).

Supporting Tables

Table S1. Sequence of primers for RT-PCR and qPCR assays used in this study. Primers were chosen from PrimerBank[2] unless otherwise referenced. Product length is shown to indicate the expected length of the PCR products amplified with RT-PCR from zebrafish RNA. bp = base pairs; F = forward; R = reverse.

Gene	Species	Primer sequence (5'-3')		Source		Product length (bp)
				PrimerBank ID	Reference	
ef1α	Zebrafish	F	CTGGTTCAAGGGATGGAAGA	—	[3]	274
		R	GAGACTCGTGGTGCATCTCA			
mpeg1	Zebrafish	F	CTTTAATTCAGAGCCACGGAGGAGC	—	[4]	526
		R	GTAGACAACCCTAAGAAACCACAGG			
mpx	Zebrafish	F	ACCAGTGAGCCTGAGACACGCA	—	[4]	488
		R	TGCAGACACCGCTGGCAGTT			
EF1α	Human	F	TGTCGTCATTGGACACGTAGA	—	[5]	—
		R	ACGCTCAGCTTTCAGTTTATCC			
IL1β	Human	F	ATGATGGCTTATTACAGTGGCAA	27894305c1	—	—
		R	GTCGGAGATTCGTAGCTGGA			
IL6	Human	F	AAGCCAGAGCTGTGCAGATGAGTA	—	[6]	—
		R	TGTCCTGCAGCCACTGGTTC			
TNFα	Human	F	CGCTCCCCAAGAAGACAG	—	[5]	—
		R	AGAGGCTGAGGAACAAGCAC			
IL12B	Human	F	ACCCTGACCATCCAAGTCAAA	24497437c1	—	—
		R	TTGGCCTCGCATCTTAGAAAG			
IL10	Human	F	TCAAGGCGCATGTGAACTCC	24430216c2	—	—
		R	GATGTCAAACTCACTCATGGCT			
MRC1	Human	F	TTCAGAAGGTTTTACTTGGAGTGA	—	[5]	—
		R	TCTCCATAAGCCCAGTTTTCA			

Supporting Movies

Movie S1. Free-circulating protocells flowing through the vasculature. Time-lapse confocal movie of protocells (green) moving, with similar pace to red blood cells, through the caudal vein at 24 h after systemic protocell injection in a 2 dpf zebrafish larva.

Movie S2. Tracking of free-circulating protocells in vasculature. Time-lapse confocal movie of protocells (green) moving through an intersegmental vessel in a 2 dpf zebrafish larva at 0.5 h after systemic protocell injection; track colors were randomly assigned to each protocell.

Movie S3. Capturing the protocell uptake by macrophages. Imaris 3D reconstruction from a time-lapse confocal movie showing a macrophage (red) uptaking a protocell (green) within the caudal artery in a 2 dpf Tg(*mpeg1:mCherry*) zebrafish larva at 0.5 h after systemic protocell injection. Note that the rotation view at the end of the movie was included to demonstrate that the protocell was fully internalized by the macrophage.

Movie S4. Protocells reside within neutrophils. Imaris 3D reconstruction from a confocal z-stack image showing a neutrophil (blue) with various internalized protocells (green) in the CHT region of a Tg(*lyz:DsRed*) larva after systemic protocell injection at 2 dpf and imaged at 24 hpi. Rotation views included to confirm that protocells were fully internalized by the neutrophil.

Movie S5. Macrophages effectively taking up free-circulating protocells from vasculature. Time-lapse confocal movie showing three consecutive uptake events of protocells (green) by macrophages (red) in a 2 dpf Tg(*mpeg1:mCherry*) zebrafish larva at 0.5 h after systemic protocell injection. Note that a single macrophage can take up multiple protocells (white arrows).

Movie S6. Neutrophils are less effective at taking up free-circulating protocells within the vasculature. Time-lapse confocal movie showing neutrophils (blue) unable to capture protocells (green) in a 2 dpf Tg(*lyz:DsRed*) zebrafish larva at 0.5 h after systemic protocell injection. Note that the concentration of protocells, imaging area, imaging starting time after protocell injection and duration of this movie were matched with Movie S5 to enable direct comparison of protocell uptake between macrophages and neutrophils.

Movie S7. Free-circulating protocells flowing through vessels at the CHT region. Time-lapse confocal movie of protocells (green) moving through the vasculature network in the CHT where a large proportion of protocells are retained within macrophages (red) in a 2 dpf Tg(*mpeg1:mCherry*) zebrafish larva at 12 h after systemic protocell injection.

Movie S8. Free-circulating protocells flowing through peripheral vessels. Time-lapse confocal movie of protocells (green) moving through an intersegmental vessel, with minimal retention in endothelial cells, in a 2 dpf zebrafish larva at 12 h after systemic protocell injection.

Movie S9. Macrophages taking up protocells after local somite injection. Time-lapse confocal movie showing how macrophages (red) are recruited and start engulfing protocells (green) at the injection site (somite) in a 3 dpf Tg(*mpeg1:mCherry*) zebrafish larva at 1.5 h after local protocell injection.

Movie S10. Macrophages taking up intact anti-miR223 protocells in fish somite. Time-lapse confocal movie showing a macrophage (dim green) engulfing protocells (bright green) loaded with anti-miR223-Cy5 (red) at the injection site (somite) in a 3 dpf Tg(*mpeg1:FRET*) zebrafish larva at 0.5 h after local protocell injection; white circles at the beginning of the movie highlight the location of protocells prior to their engulfment by the macrophage.

Movie S11. Capturing the protocell uptake by human macrophages. Imaris 3D reconstruction from time-lapse confocal movie showing the uptake of a protocell (green) by a human macrophage (red) in vitro 2 h after protocell

supplementation. Note that the rotation view at the end of the movie was included to demonstrate that the protocell was fully internalized by the macrophage.

Supporting References

[1] M. O. Koch, MD, J. K. Daggy, MS, T. M. Ulbright, MD, J. N. Eble, MD, L. Cheng, MD, L. E. Eichelberger, MD, *Am. J. Clin. Pathol.* **2003**, *120*, 386.
[2] A. Spandidos, X. Wang, H. Wang, B. Seed, *Nucleic Acids Res.* **2010**, *38*, D792.
[3] Y. Feng, C. Santoriello, M. Mione, A. Hurlstone, P. Martin, *PLoS Biol.* **2010**, *8*, e1000562.
[4] W. Zhou, A. S. Pal, A. Y.-H. Hsu, T. Gurol, X. Zhu, S. E. Wirbisky-Hershberger, J. L. Freeman, A. L. Kasinski, Q. Deng, *Cell Rep.* **2018**, *22*, 1810.
[5] D. B. Gurevich, C. E. Severn, C. Twomey, A. Greenhough, J. Cash, A. M. Toye, H. Mellor, P. Martin, *EMBO J.* **2018**, *37*, e97786.
[6] S. Shinriki, H. Jono, K. Ota, M. Ueda, M. Kudo, T. Ota, Y. Oike, M. Endo, M. Ibusuki, A. Hiraki, H. Nakayama, Y. Yoshitake, M. Shinohara, Y. Ando, *Clin. Cancer Res.* **2009**, *15*, 5426.
